# Development and validation of the guideline for reporting evidence-based practice educational interventions and teaching (GREET)

**DOI:** 10.1186/s12909-016-0759-1

**Published:** 2016-09-06

**Authors:** Anna C. Phillips, Lucy K. Lewis, Maureen P. McEvoy, James Galipeau, Paul Glasziou, David Moher, Julie K. Tilson, Marie T. Williams

**Affiliations:** 1 School of Health Sciences, University of South Australia, GPO box 2471, Adelaide, 5001 Australia; 2 Sansom Institute for Health Research, Alliance for Research in Exercise, Nutrition and Activity (ARENA), University of South Australia, Adelaide, Australia; 3 Faculty of Medicine, Nursing and Health Sciences, School of Health Sciences, Flinders University, Adelaide, Australia; 4 Ottawa Hospital Research Institute, Centre for Practice-Changing Research (CPRC), The Ottawa Hospital, 501 Smyth Rd, Ottawa, ON K1H 8L6 Canada; 5 Centre for Research in Evidence-Based Practice (CREBP), Bond University, University Drive, Robina, QLD 4226 Australia; 6 Ottawa Hospital Research Institute, Clinical Epidemiology Program, Centre for Practice-Changing Research (CPCR), The Ottawa Hospital, 501 Smyth Rd, Ottawa, ON K1H 8L6 Canada; 7 Division of Biokinesiology and Physical Therapy, University of Southern California, 1540 Alcazar St, CHP155, Los Angeles, 90089 USA

**Keywords:** Evidence-based practice, Education, Reporting guideline, Validation, Research design

## Abstract

**Background:**

The majority of reporting guidelines assist researchers to report consistent information concerning study design, however, they contain limited information for describing study *interventions*. Using a three-stage development process, the Guideline for Reporting Evidence-based practice Educational interventions and Teaching (GREET) checklist and accompanying explanatory paper were developed to provide guidance for the reporting of educational interventions for evidence-based practice (EBP). The aim of this study was to complete the final development for the GREET checklist, incorporating psychometric testing to determine inter-rater reliability and criterion validity.

**Methods:**

The final development for the GREET checklist incorporated the results of a prior systematic review and Delphi survey. Thirty-nine items, including all items from the prior systematic review, were proposed for inclusion in the GREET checklist. These 39 items were considered over a series of consensus discussions to determine the inclusion of items in the GREET checklist. The GREET checklist and explanatory paper were then developed and underwent psychometric testing with tertiary health professional students who evaluated the completeness of the reporting in a published study using the GREET checklist. For each GREET checklist item, consistency (%) of agreement both between participants and the consensus criterion reference measure were calculated. Criterion validity and inter-rater reliability were analysed using intra-class correlation coefficients (ICC).

**Results:**

Three consensus discussions were undertaken, with 14 items identified for inclusion in the GREET checklist. Following further expert review by the Delphi panelists, three items were added and minor wording changes were completed, resulting in 17 checklist items. Psychometric testing for the updated GREET checklist was completed by 31 participants (*n* = 11 undergraduate, *n* = 20 postgraduate). The consistency of agreement between the participant ratings for completeness of reporting with the consensus criterion ratings ranged from 19 % for item 4 Steps of EBP, to 94 % for item 16 Planned delivery. The overall consistency of agreement, for criterion validity (ICC 0.73) and inter-rater reliability (ICC 0.96), was good to almost perfect.

**Conclusion:**

The final GREET checklist comprises 17 items which are recommended for reporting EBP educational interventions. Further validation of the GREET checklist with experts in EBP research and education is recommended.

**Electronic supplementary material:**

The online version of this article (doi:10.1186/s12909-016-0759-1) contains supplementary material, which is available to authorized users.

## Background

The underlying basis of educational interventions is to increase learners’ competence and skills in a specific content area and to promote lifelong learning [1]. Evidence-based practice (EBP) is a decision making paradigm in health care that integrates the patient’s perspective, practitioner expertise and the best available research evidence [2]. Education in the principles and practice of EBP is widely accepted as a core component of professional education for healthcare professionals [3, 4]. However, the most effective teaching strategies for promoting the effective use of EBP in practice are uncertain [1]. Inconsistent reporting of interventions used in EBP educational research is a significant barrier to identifying the most effective teaching strategies [1, 5–7]. Many studies investigating EBP educational interventions provide insufficient details about the educational intervention, limiting interpretation, synthesis in secondary research, and replication [1].

In 1994, in an attempt to address the ‘wide chasm’ between what a randomized controlled trial (RCT) *should* report and what is *actually* reported, the Standards of Reporting Trials Group developed a ‘proposal for the structured reporting of RCT’s’ [8]. This proposal later became the CONSORT statement (CONsolidated Standards Of Reporting Trials), which is one of the earliest and most well-established reporting guidelines [9]. The CONSORT statement led the way for the development of a multitude of reporting guidelines in the form of checklists, flow diagrams and explicit instructional papers providing guidance for authors reporting a variety of research designs [10, 11].

Over the past two decades, reporting guidelines for study designs have assisted researchers, authors and reviewers in providing consistent and explicit information concerning study design. However, limited information is available in these design-specific reporting guidelines for describing details of the *interventions* within studies [12]. Educational interventions are complex and it is not always possible or appropriate for an educational intervention to follow a strict formula such as in a pharmaceutical intervention [12, 13]. As educational interventions frequently require modifications to ensure that they meet the needs of the learner, detailed reporting of the intervention is vital to enable replication [12, 13].

To date, there are five reporting guidelines listed on the EQUATOR network that are specifically focused on describing educational interventions [14–18], but there are no guidelines specifically for EBP educational interventions. Therefore, we developed a reporting guideline to guide educators and researchers reporting educational interventions designed to develop EBP learning [19]. This guideline is called the Guideline for Reporting Evidence-based practice Educational interventions and Teaching (GREET), and is comprised of a checklist, and accompanying explanation and elaboration (E&E) paper.

The first stages of the development procedure for the GREET checklist and E&E paper have been published elsewhere [19–21]. Preliminary testing is recommended as part of the development process for a reporting guideline [10]. Therefore, the aim of this study was to describe the final development for the GREET checklist and E&E paper, incorporating psychometric testing to determine inter-rater reliability and criterion validity.

## Methods

### Development of the GREET checklist and the E&E paper

Development of the GREET checklist was prospectively planned to follow the Guidance for Developers of Health Research Reporting Guidelines [10]. The original protocol for development of the GREET checklist incorporated three broad stages [19]; 1) systematic review [19], 2) consensus processes including Delphi survey [20]; and 3) development and pilot testing for the GREET checklist and the E&E paper [19]. At the time of development of the GREET checklist, a reporting guideline was being developed as a generic guide for describing interventions Template for Intervention Description and Replication’ (TIDieR) [12]. In order to ensure consistency between these reporting guidelines, the TIDieR framework was adopted as a starting point for the GREET checklist.

Several teams were involved in the development of the GREET checklist. The research team consisted of a doctoral and expert panel. The doctoral panel comprised of the principal investigator (AP) undertaking this research as part of a Doctor of Philosophy in Health Science (PhD), and the supervisory team (MTW, LKL, MPM). The expert panel was comprised of five members who were invited due to their prior knowledge and experience in EBP educational theory and research, development of reporting guidelines and the dissemination of scientific information (JG, PG, MH, DM, JKT). As part of Stage 2 of development of the GREET checklist, international experts in EBP and research reporting participated in the Delphi survey (Delphi panelists) [21].

**Fig. 1:**
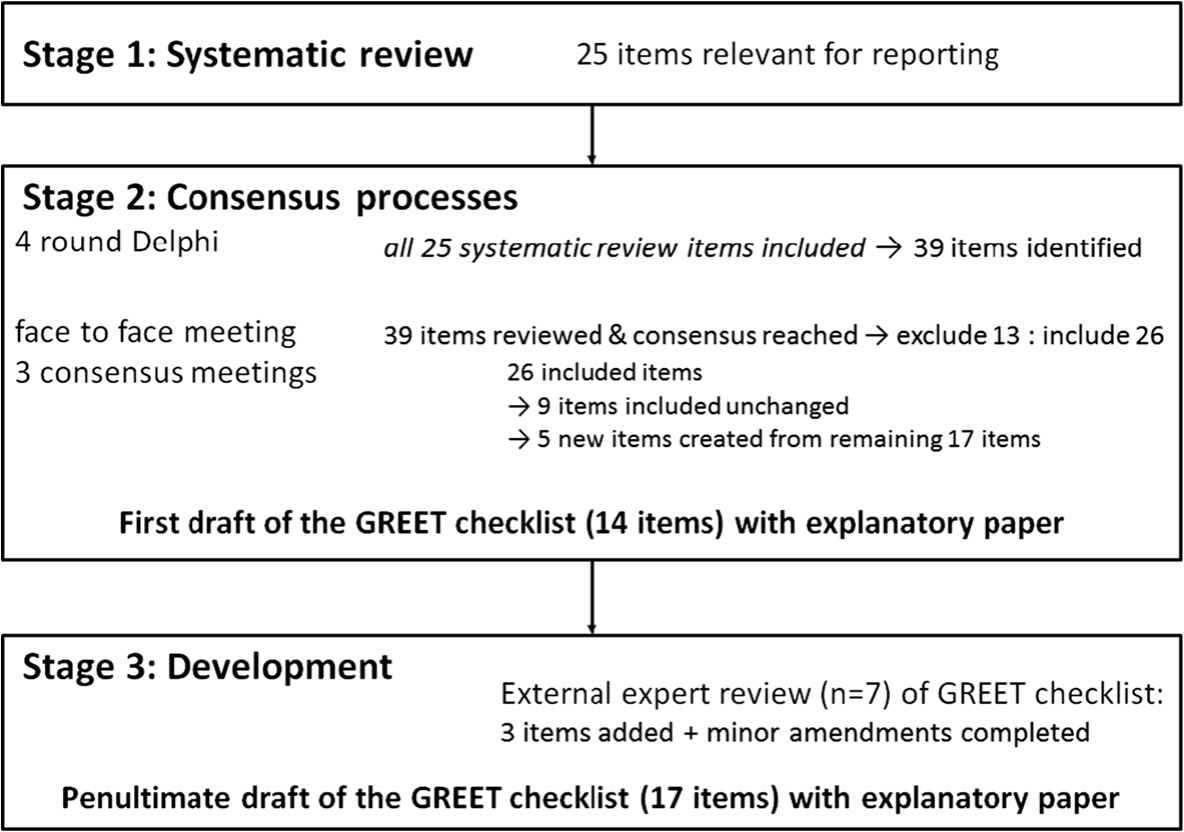
Summary of the three development stages for the GREET checklist including those completed prior to the psychometric testing

The first stage in the development process for the GREET checklist comprised a systematic review which identified 25 items relevant for reporting an EBP educational intervention (Fig. 1) [20]. To ensure all 25 items identified in the systematic review were included in the next stage of the development process (the four round Delphi survey), cross checking of these 25 items was completed at the end of the second round of the Delphi survey [21]. Six items identified in the systematic review that were not included in the Delphi list at the completion of the second round were added as ‘additional’ items for the third round of the Delphi. Hence rounds’ 3 and 4 included items derived from Delphi participants and items derived from the systematic review. At the completion of the four round Delphi survey, 39 items were nominated for consideration for describing an educational intervention for developing foundation knowledge and skills of EBP [21].

To determine the final inclusion of items for the GREET checklist, further consensus activities were undertaken. These comprised a series of consensus meetings via international teleconference with the research team. Three international teleconferences were required to attain a consensus decision (Table 1).

**Table 1:** Overview of the three international consensus teleconferences

	Consensus 1	Consensus 2	Consensus 3
Date	18/6/2013	30/7/13	20/8/13
Time commenced	2100 AEST	2100 AEST	2100 AEST
	0700 Canada	0700 Canada	0700 Canada
	1200 United Kingdom	1300 France	1300 France
Attendees *n* (%)	7(88)	8 (100)	7 (88)
	AP, MTW, MPM, JG, PG, DM, MH	AP, MTW, MPM, LKL, JG, PG, DM, MH	AP, MTW, MPM, LKL, PG, DM, MH
Duration (mins)	85	73	82
Agenda	Review of Delphi intervention items 1–24.	Review of remaining 15 Delphi intervention items 25–39.	Review of wording for the GREET checklist items.
			Discuss options for pilot testing the GREET.
Outcomes	Include: 13 items	Include: 13 items	No wording changes: 8 items
	Exclude: 11 items	Exclude: 2 items	Wording changes: 6 items
Post meeting activities	Reword and combine 9 of 13 included items.	Reword and combine items.	Complete rewording for the GREET items and first complete draft of checklist. Draft and submit proposal for pilot testing to ethics.
		Prepare first full list of items for inclusion in the GREET checklist.	

*AEST* australian eastern standard time, *AP* Anna Phillips, *MTW* Marie Williams, *MPM* Maureen McEvoy, *JG* James Galipeau, *PG* Paul Glasziou, *DM* David Moher, *MH* Marilyn Hammick, *LKL* Lucy Lewis

Over the course of the international consensus teleconferences, all 39 intervention items arising from the Delphi survey were reviewed, with consensus agreement (majority vote) to retain 26 (67 %) items essential for reporting and to omit 13 (33 %) non-essential items. Following the agreed refinement of the 26 retained items, 14 items were included in the first draft of the GREET checklist (Fig. 1).

The first draft of the GREET checklist underwent further review by seven Delphi panelists who had previously indicated their willingness to provide feedback. As a result of this review, three additional checklist items were included (‘Incentives’; ‘Learner time spent in face-to face contact with instructor/self-directed learning activities’; ‘Extent to which the intervention was delivered as scheduled’). Therefore, the penultimate GREET checklist comprised 17 items recommended for the reporting educational interventions for EBP.

The GREET checklist was intended to be used in conjunction with an E&E paper to provide guidance and instructions for users. Following completion of the penultimate GREET checklist, the E&E paper was developed using a standard framework for each item; this included an explanation for each GREET checklist item along with the relevance of the item for reporting, verbatim examples of explicit reporting from previously published studies and a troubleshooting section to assist in resolving uncertainties or potentially confusing issues.

### Inter-rater reliability and criterion validity

#### Design

An observational cross-sectional design was used to assess inter-rater reliability and criterion validity of the GREET checklist among readers of an EBP educational intervention study.

### Participants

A sample of tertiary health professional students in the final years of their programs (physiotherapy, podiatry, medical radiation, human movement, occupational therapy, population health, dietetics) and postgraduate health research students (PhD) in the Division of Health Sciences at the University of South Australia were recruited towards the end of a semester to avoid conflicts with academic or final examination commitments. Participants were provided with an AUD30 gift card as compensation for their time. There was a mix of participants with and without prior experience in EBP education or reporting guidelines.

### Procedure

Participants were invited to read a published study and to indicate whether and where each of the items in the GREET checklist were reported. The E&E paper was provided to participants for clarification of items as needed.

### Test study identification

To identify an appropriate research study for participants to review, the search strategy from our previous systematic review [20] was re-run on 22^nd^ November 2013 to identify papers published since the original search was undertaken. All recent studies meeting the original eligibility criteria were reviewed by the doctoral panel for relevance and reporting using the draft GREET checklist. The test study [22] was selected by consensus agreement by the doctoral team as the most appropriate test study, including a wide range in the completeness and level of detail of the reporting.

To enable comparison of ratings of completeness of reporting provided by participants, a criterion reference measure was developed. All members of the doctoral panel (AP, LKL, MPM, MTW) independently evaluated the test study [22] using the GREET checklist and the E&E paper and then met to discuss each of ratings assigned for the items in the GREET checklist. The final ratings assigned to each item in GREET checklist for the criterion reference measure were determined by consensus agreement of the doctoral panel.

### Validation process

The testing process used an experiential learning approach [23], and consisted of two parts; participants were required: 1) to use the GREET checklist (+/-E&E paper) to review the test study [22] and to indicate whether each checklist item was reported using possible responses [(1) Yes- fully reported, (2) Yes- partially reported, (3) No- not reported or No- not clear], and 2) to provide comment and rate the ease of use for each item. Participants were also invited to provide feedback regarding the wording and layout for individual items and for the overall GREET checklist and the E&E paper. Comments on participants’ experience of the validity-testing process were also sought.

All testing was undertaken in small groups (1–7 participants) and supervised by the principal investigator (AP), with each participant completing the process independently on a computer. This ensured that any questions or problems encountered during the testing process could be noted and addressed appropriately. At the commencement of each two hour session, a standardised overview of the procedure was presented and participants were provided with hard copies of the GREET checklist, the E&E paper and the test study [22].

### Data collection tool

Data collection was undertaken using an online instrument (SurveyMonkey®) which was pilot tested by three members of the doctoral team (MTW, MPM, LKL) prior to the psychometric testing. In section 1, participants were invited to provide basic demographic data including their age, gender, study discipline, previous exposure to education in EBP and experience using reporting guidelines.

In section 2, for each item in the GREET checklist, participants were asked to indicate whether they perceived the item to be reported in the test study (Yes- fully reported, Yes- partially reported, No- not reported or No-not clear). If the item was reported, participants were invited to extract and document verbatim information that was relevant to the specific item. For each checklist item, participants were requested to indicate whether they used the GREET checklist alone or in conjunction with the E&E paper when making a decision about whether information was reported for that item in the test study. Participants were then asked to provide a Yes/No rating for the ease of use related to layout and wording of the specific item in the GREET checklist and in the E&E paper and space was provided for suggested re-wording. In Section 3, participants rated their experience using the GREET checklist and E&E paper on a 5-point Likert scale ranging from 1: poor to 5: excellent. Space was provided for any further comments.

### Data analysis

Data from all completed surveys were downloaded to a spread sheet (Excel. Version 14. Microsoft; 2010). Demographic data were collated and summarised. Participant responses for completeness of reporting for each of the GREET checklist items were allocated into one of three categories, (1) YES fully reported, (2) YES partially reported and (3) NO not reported, or NO not clear. These ratings were summarised descriptively according to agreement between participants and with the consensus criterion standard. The level of agreement was specified as “agreement” where there was exact agreement of the participant rating with the consensus criterion rating (both ratings of agreement in the same category), “partial agreement” (one category of difference between the participant rating and the consensus criterion rating) or “no agreement” (two categories of difference between the participant rating and the consensus criterion rating). Percentage agreement for these categories were calculated. The consistency of agreement for the participants’ ratings of completeness of reporting with the consensus criterion standard (criterion validity) and agreement for the ratings of completeness of reporting between participants (inter-rater reliability) were analysed using intra-class correlation coefficients (ICC) (two-way mixed, consistency, average-measures ICC) (IBM SPSS statistics 21). The ICC coefficients were interpreted based on the recommendations by (Landis & Koch [24], p165), where the level of agreement indicated by the ICC values of less than zero = poor, 0 to 0.2 = slight, 0.21 to 0.4 = fair, 0.41 to 0.6 = moderate, 0.61 to 0.8 = strong and greater than 0.8 = almost perfect agreement.

The rating for the ease of use related to layout and wording of the specific item for the GREET checklist and the E&E paper were summarised descriptively. Chi square tests (χ^2^) were undertaken to analyse differences between those with previous experience of EBP training or exposure to reporting guidelines. Statistical significance was set at *p* < 0.05.

## Results

### Participants

Testing of the GREET checklist and the E&E paper was completed by 31 participants (*n* = 11 undergraduate, *n* = 20 postgraduate) during nine, two hour sessions. Participant demographic data are shown in Table 2.

**Table 2:** Participant demographic information

Demographic information	*n* (%)
Gender	
Female	22 (71)
Male	9 (29)
Age (years) Mean (SD)	34 (12), range 20–59
Professional role	
Undergraduate student	11 (36)
Post graduate student	17 (55)
Researcher and teacher	2 (6)
Researcher	1 (3)
Professional discipline	
Physiotherapy	15 (47)
Population Health	4 (12)
Epidemiology	2 (7)
Human Movement	2 (7)
Medical Radiations	2 (7)
Occupational therapy	2 (7)
Dietetics	2 (7)
Education	1 (3)
Podiatry	1 (3)
Previous EBP education or training	
Yes	22 (71)
No	9 (29)
Previous exposure to reporting guidelines	
Yes	17 (55)
No	14 (45)
English first language	
Yes	28 (90)
No	3 (10)
Time (mins) (SD) to complete testing	77 (23)

### Criterion validity and inter-rater reliability

All participants rated completeness of reporting for each of the GREET checklist items using the test study (i.e. no missing data) (Additional file 1). The consistency of the agreement of participants ratings for the completeness of the reporting of each item in the GREET checklist with consensus criterion ratings are presented in Table 3.

**Table 3:** Summary of consistency of agreement for participants ratings for completeness of the test study’s reporting for items in the GREET checklist with consensus criterion ratings

The GREET checklist item	*n*	Agreement with consensus criterion ratings		
		Agreement^a^	Partial^a^ agreement	No^a^ agreement
		*n* (%)	*n* (%)	*n* (%)
1. Title	31	21 (68)	8 (26)	2 (6)
2. Theory	31	18 (58)	7 (23)	6 (19)
3. Learning objectives	31	18 (58)	11 (35)	2 (6)
4. Steps of EBP	31	6 (19)	18 (58)	7 (23)
5. Materials	31	8 (26)	20 (65)	3 (9)
6. Learning strategies	31	22 (71)	8 (26)	1 (3)
7. Incentives	31	24 (78)	1 (3)	6 (19)
8. Instructors	31	25 (81)	6 (19)	0 (0)
9. Delivery	31	16 (52)	10 (32)	5 (16)
10. Environment	31	15 (49)	9 (28)	7 (23)
11. Schedule	31	16 (52)	14 (45)	1 (3)
12. Face to face time	31	18 (58)	8 (26)	5 (16)
13. Adaptations	31	16 (52)	9 (28)	6 (20)
14. Modifications	31	26 (84)	1 (3)	4 (13)
15. Attendance	31	20 (64)	7 (23)	4 (13)
16. Planned delivery	31	29 (94)	0 (0)	2 (6)
17. Actual schedule	31	25 (81)	4 (13)	2 (6)
Reliability:	ICC (95 % CI), *p*=			
Criterion validity ICC (95 % CI), *p*=	0.73 (.51–.88), *p* < .0001			
Inter-rater reliability ICC (95 % CI), *p*=	0.96 (.93–.98), *p* < .0001			

*ICC* intra class correlation coefficient

^a^The participants’ ratings for completeness of reporting were (1) Yes- fully reported, (2) Yes- partially reported, (3) No- not reported or No- not clear. Consistency of agreement with the consensus criterion ratings was defined as: Agreement (both the participant ratings of agreement and the consensus criterion rating were in the same category), Partial agreement (one category of difference between the participant rating and the consensus criterion rating) or No agreement (two categories of difference between the participants rating with the consensus criterion rating)

The consistency of agreement between the participant ratings for completeness of reporting with the consensus criterion ratings (the participants ratings and the consensus criterion ratings were exactly the same) ranged from 19 % for item 4 Steps of EBP to 94 % for item 16 Planned delivery. Four items showed the greatest agreement between the participants’ ratings for completeness of reporting and the consensus criterion ratings, 16 Planned delivery (94 %), 14 Modifications (84 %), 17 Actual schedule (81 %) and 8 Instructors (81 %) (Table 3). The items with the greatest difference between the participants ratings for completeness of reporting and the consensus criterion ratings (2 categories of difference in the ratings) were : 4 Steps of EBP (23 %), 10 environment (23 %) and 13 Adaptations (20 %), with no agreement between 20 to 23 % of participants ratings for completeness of reporting and the consensus criterion ratings (Table 3).

Overall, consistency of agreement between participants’ ratings of completeness of reporting and the consensus criterion ratings was strong (criterion validity ICC 0.73, 95 % CI 0.51–0.88, *p* <0 .0001). There was almost perfect consistency of agreement within the participant group for ratings of completeness (inter-rater reliability) (ICC 0.96, 95 % CI 0.93–0.98, *p* < 0.0001) (Table 3).

### Participant ratings for the wording of items in the GREET checklist

The majority of participants (97 to 100 %) provided a yes/no response to the question “Did you find the wording and layout easy to use for this item in the GREET checklist?” Six items, (7 Incentives, 8 Instructors, 10 Environment, 11 Schedule, 12 Face to face time, 14 Modifications) were rated by all participants as “yes”- the wording and layout was easy to use. Item 1 intervention, achieved the least number of “yes” ratings (70 % of participants).

An overall rating for the layout and ease of use of the GREET checklist was provided by the majority of participants (*n* = 30, 97 %). The ratings, on a 5-point scale from poor to excellent, were positive, with participants rating the overall layout and ease of use as Poor (*n* = 0), Fair (*n* = 2, 7 %), Good (*n* = 12, 40 %), Very Good (*n* = 12, 40 %), and Excellent (*n* = 4, 13 %).

### Evaluation of the E&E paper

The E&E paper was used inconsistently, with participants indicating that they selectively referred to this depending upon the checklist item; less than half (48 %) of the participant group referred to the E&E paper for item 8 Instructors compared to the majority of participants (87 %) for item 2 Theory. Participants, who referred to the E&E paper, rated the E&E paper positively, with 75 % of participants rating all items as easy to use.

### Prior experience with EBP or reporting guidelines

Participants did not rate the layout and ease of use of the GREET checklist significantly differently based on prior exposure to EBP training (χ^2^ = 3.41, *p* = 0.33) or experience with reporting guidelines (χ^2^ = 4.22, *p* = 0.24). No significant difference was found between the level of agreement with the consensus criterion ratings for any of the GREET checklist items based on prior exposure to EBP training or experience with reporting guidelines.

### Participant comments

Participants provided 185 separate comments regarding the wording and layout of the items in the GREET checklist and the E&E paper. The majority of these comments (*n* = 105, 57 %) concerned reinforcing or justifying their ratings for whether the item was reported in the study. For example: “*The learning objectives were very detailed and I was unsure how much detail to provide.*”

A small number of comments were provided for re-wording items in the GREET checklist (*n*=15, 16 %) and the E&E paper (*n* = 14, 16 %). For example: “*I think the BRIEF NAME/TITLE, initially makes me refer to the title of the study. However, the GREET checklist description to provide the educational intervention requires further reading from the article. I would think INTERVENTION is a better heading.”*

### Outcomes for the GREET checklist

The GREET checklist and the E&E paper were updated based on participant ratings for whether the wording and layout were easy to use, the agreement for the participants’ ratings for completeness of reporting with the consensus criterion and between the raters, and the comments and suggestions provided by the participants. Wording changes were made to eight items; 1 Title (changed to Intervention), 3 Learning Objectives (added all groups involved), 4 Steps of EBP (changed to EBP content), 6 Learning Strategies (changed to Educational Strategies), 13 Adaptations (reworded to Planned changes), 16 Planned delivery (further information added to describe materials and educational strategies) and 17 Actual Schedule (actual schedule removed from heading and further information provided regarding the planned schedule of the intervention) (Table 4).

**Table 4:** The GREET checklist 2016^a^

BRIEF NAME
1. INTERVENTION: Provide a brief description of the educational intervention for all groups involved [e.g. control and comparator(s)].
WHY - this educational process
2. THEORY: Describe the educational theory (ies), concept or approach used in the intervention.
3. LEARNING OBJECTIVES: Describe the learning objectives for all groups involved in the educational intervention.
4. EBP CONTENT: List the foundation steps of EBP (ask, acquire, appraise, apply, assess) included in the educational intervention.
WHAT
5. MATERIALS: Describe the specific educational materials used in the educational intervention. Include materials provided to the learners and those used in the training of educational intervention providers
6. EDUCATIONAL STRATEGIES: Describe the teaching/learning strategies (e.g. tutorials, lectures, online modules) used in the educational intervention.
7. INCENTIVES: Describe any incentives or reimbursements provided to the learners.
WHO PROVIDED
8. INSTRUCTORS: For each instructor(s) involved in the educational intervention describe their professional discipline, teaching experience/expertise. Include any specific training related to the educational intervention provided for the instructor(s).
HOW
9. DELIVERY: Describe the modes of delivery (e.g. face-to-face, internet or independent study package) of the educational intervention. Include whether the intervention was provided individually or in a group and the ratio of learners to instructors.
WHERE
10. ENVIRONMENT: Describe the relevant physical learning spaces (e.g. conference, university lecture theatre, hospital ward, community) where the teaching/learning occurred.
WHEN and HOW MUCH
11. SCHEDULE: Describe the scheduling of the educational intervention including the number of sessions, their frequency, timing and duration.
12. Describe the amount of time learners spent in face to face contact with instructors and any designated time spent in self-directed learning activities.
PLANNED CHANGES
13. Did the educational intervention require specific adaptation for the learners? If yes, please describe the adaptations made for the learner(s) or group(s).
UNPLANNED CHANGES
14. Was the educational intervention modified during the course of the study? If yes, describe the changes (what, why, when, and how).
HOW WELL
15. ATTENDANCE: Describe the learner attendance, including how this was assessed and by whom. Describe any strategies that were used to facilitate attendance.
16. Describe any processes used to determine whether the materials (item 5) and the educational strategies (item 6) used in the educational intervention were delivered as originally planned.
17. Describe the extent to which the number of sessions, their frequency, timing and duration for the educational intervention were delivered as scheduled (item 11).

^a^based on the TIDieR guidance [12]

We strongly recommend reading this statement in conjunction with the GREET 2016 explanation and elaboration paper for important clarifications on all the items. If relevant, we also recommend reading the TIDieR guidance [12]

The final, complete version E&E paper is provided as an online supplement (Additional file 2).

### Limitations

The original plan to test the GREET checklist and the E&E paper was to have Delphi panelists trial the GREET checklist and the E&E paper during the writing phase for a manuscript or recent educational intervention for EBP. In the final round of the Delphi survey, participants were asked ‘Would you be interested in reviewing the draft of the reporting guideline and associated document?’ and ‘If you are currently undertaking an EBP educational strategy and plan to submit this for publication, would you be willing to pilot test the draft guideline.’ None of the Delphi participants accepted this invitation. As such, the psychometric testing of the GREET checklist has several limitations. Firstly, in this validation study, appraisal of reporting rather than use of the checklist to guide reporting a paper was tested. Rather than researchers and educators experienced in EBP, the study sample included a range of non-expert users with varying experience and exposure to EBP education and reporting guidelines. Secondly, the sample size was small and participants were recruited from one tertiary institution. Although there were a variety of health professions represented, there were no medical or nursing students and these groups are widely represented in authorship of studies investigating educational interventions for EBP. Thirdly, the role of the criterion standard was to provide a reference point by which to compare the ratings provided by the participants. However, as the criterion ratings were based on consensus, they reflected the opinion of the doctoral panel, rather than a set of ‘correct’ ratings. Finally, inclusion of the response category “NO not reported, or NO not clear” created an ambiguous response option, similar to “neutral”, or “neither agree nor disagree” option. These categories may have been used by respondents to indicate a range of options (lack of clarity in the question, unable to confidently attribute to a different response option or inadequate knowledge of the question content).

## Discussion

This study aimed to complete the final development for the GREET checklist, incorporating provisional psychometric testing to determine inter-rater reliability and criterion validity.

Although the consistency of agreement, both between the participants and between the participants and the consensus criterion standard, was good to almost perfect, the consistency of agreement in the ratings between participants (ICC 0.96) was considerably higher than between participants and the consensus criterion ratings (ICC 0.73) [24]. It is possible the lower overall consistency of agreement for the criterion validity was a result of the differences in expertise and experience of the participants compared with the doctoral team responsible for the consensus criterion ratings. Collectively, the doctoral team were experienced in EBP education, terminology and study appraisal, whereas more than one quarter of the participants had no previous experience or training in EBP. For all participants, this was their first exposure to the GREET checklist and for almost half of the participants their first exposure to a reporting guideline. The differences in expertise and experience with EBP and reporting guidelines may also provide an explanation for the difference between the participants’ ratings for completeness of reporting and the consensus criterion ratings for the items, 4 Steps of EBP and 5 Materials, where almost one quarter of participant ratings had no agreement with the consensus criterion ratings.

The purpose of the GREET checklist and the E&E paper is to provide specific guidance for the reporting of EBP educational interventions, rather than to replicate reporting guidelines for specific research designs. As such, the GREET checklist and the E&E paper were designed to be used in conjunction with an appropriate existing reporting guideline for the study design.

While it may seem burdensome to add further reporting guidelines for interventions to the already long list of items required in current guidelines for study design, the consistent and transparent reporting of interventions in primary educational research studies is just as important. For educators applying research into their teaching, and for consumers of research, reporting guidelines can provide structure for interpreting the relevance of information, and a method of identifying possible biases in the reporting of interventions.

## Conclusion

The GREET checklist is a reporting guideline designed to provide a framework for the consistent and transparent reporting for educational interventions for EBP. Used together with the E&E paper, developed to enhance its use and understanding, the GREET checklist could further facilitate development of an evidence-base for EBP education. Further targeted, user-specific, review and validation of the GREET checklist with experts in EBP research and education is recommended.

## Additional files

Additional file 1: Dataset for psychometric testing of the GREET checklist. Complete dataset with results of participant responses (*n* = 31) for completeness of reporting for each item in the GREET checklist, ratings for whether the GREET checklist and E&E paper were easy to use and all comments provided by participants for the psychometric testing of the GREET checklist. (XLSX 82 kb) Additional file 2: 2016 Explanation and elaboration paper for the GREET checklist. An explanation and elaboration document developed to be used in conjunction with the GREET checklist to enhance the use and understanding for the information items in the GREET checklist. (DOCX 130 kb)

## Abbreviations

AP: Anna Phillips
CONSORT: CONsolidated standards of reporting trials
DM: David Moher
E&E: Explanation and elaboration paper
EBP: Evidence-based practice
GREET: Guideline for reporting evidence-based practice educational interventions and teaching
ICC: Intra-class correlation coefficient
JG: James Galipeau
JKT: Julie K Tilson
LKL: Lucy K Lewis
MPM: Maureen P McEvoy
MTW: Marie T Williams
MH: Marilyn Hammick
PhD: Doctor of Philosophy
RCT: Randomized controlled trial
TIDieR: Template for intervention description and replication
