# Supplementary material for: Development and validation of the guideline for reporting evidence-based practice educational interventions and teaching (GREET)
Source: BMC Med Educ. 2016 Sep 6;16(1):237. doi: 10.1186/s12909-016-0759-1 (PMC5011880; doi:10.1186/s12909-016-0759-1)
Supplement: Additional file 2: — 2016 Explanation and elaboration paper for the GREET checklist. An explanation and elaboration document developed to be used in conjunction with the GREET checklist to enhance the use and understanding for the information items in the GREET checklist. (DOCX 130 kb) [file 12909_2016_759_MOESM2_ESM.docx]

| 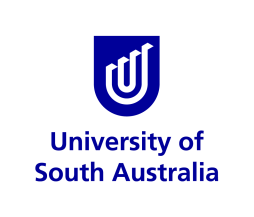 | **Explanation and elaboration paper (E&E)**  **for the Guideline for Reporting Evidence-based practice Educational interventions and Teaching (GREET).** |
| --- | --- |

**2016**

Developed by:

Ms Anna Phillips, Dr Lucy Lewis, Dr Maureen McEvoy, Dr James Galipeau, Dr Paul Glasziou, Dr Marilyn Hammick, Dr David Moher, Dr Julie Tilson and Dr Marie Williams.

| **Table of contents** | **page** | |
| --- | --- | --- |
| Introduction | **3** |  |
| How to report an educational intervention for evidence-based practice (EBP) | **4** |  |
| Explanation for checklist items 1-17 | **5-17** |  |
| **Item 1 INTERVENTION:** Provide a brief description of the educational intervention for all groups involved [e.g. control and comparator(s)]. | **5** |  |
| **Item 2 THEORY**: Describe the educational theory (ies), concept or approach used in the intervention. | **5** |  |
| **Item 3 LEARNING OBJECTIVES**: Describe the learning objectives for all groups involved in the educational intervention. | **6** |  |
| **Item 4 EBP CONTENT**: List the foundation steps of EBP (ask, acquire, appraise, apply, assess) included in the educational intervention. | **7** |  |
| **Item 5 MATERIALS**: Describe the specific educational materials used in the educational intervention. Include materials provided to the learners and those used in the training of educational intervention providers. | **8** |  |
| **Item 6** **EDUCATIONAL STRATEGIES**: Describe the teaching / learning strategies (e.g. tutorials, lectures, online modules) used in the educational intervention. | **9** |  |
| **Item 7 INCENTIVES**: Describe any incentives or reimbursements provided to the learners. | **9** |  |
| **Item 8 INSTRUCTORS**: For each instructor(s) involved in the educational intervention describe their professional discipline, teaching experience / expertise. Include any specific training related to the educational intervention provided for the instructor(s). | **10** |  |
| **Item 9 DELIVERY**: Describe the modes of delivery (e.g. face-to-face, internet or independent study package) of the educational intervention. Include whether the intervention was provided individually or in a group and the ratio of learners to instructors. | **11** |  |
| **Item 10 ENVIRONMENT**: Describe the relevant physical learning spaces (e.g. conference, university lecture theatre, hospital ward, community) where the teaching / learning occurred. | **12** |  |
| **Item 11 SCHEDULE**: Describe the scheduling of the educational intervention including the number of sessions, their frequency, timing and duration. | **12** |  |
| **Item 12** Describe the amount of time learners spent in face to face contact with instructors and any designated time spent in self-directed learning activities. | **14** |  |
| **Item 13** Did the educational intervention require specific adaptation for the learners? If yes, please describe the adaptations made for the learner(s) or group(s). | **14** |  |
| **Item 14** Was the educational intervention modified **during** the course of the study? If yes, describe the changes (what, why, when, and how). | **15** |  |
| **Item 15 ATTENDANCE**: Describe the learner attendance, including how this was assessed and by whom. Describe any strategies that were used to facilitate attendance | **15** |  |
| **Item 16** Describe any processes used to determine whether the materials (item 5) and the educational strategies (item 6) used in the educational intervention were delivered as originally planned**.** | **16** |  |
| **Item 17** Describe the extent to which the number of sessions, their frequency, timing and duration for the educational intervention was delivered as scheduled (item 11). | **17** |  |
| Conclusion | **18** |  |
| The GREET checklist | **19** |  |
| References | **20-25** |  |

**Introduction**

This explanation and elaboration (E&E) paper has been designed to accompany the Guideline for Reporting Evidence-based practice Educational interventions and Teaching (GREET) checklist. The purpose of this E&E paper is to enhance the use, understanding and dissemination of the GREET checklist [1].

The GREET checklist includes items recommended for reporting educational interventions for facilitating foundation knowledge and skills in evidence-based practice (EBP). The GREET checklist is intended as a guide for authors to help in the planning phase for an educational intervention for EBP, researchers designing an educational intervention for EBP, as a guide for the preparation of a report for publication purposes, and potentially, as a template for appraising studies reporting an educational intervention for EBP. While the GREET checklist has been developed for educational interventions for EBP, the checklist may potentially be useful as a generic guide for reporting educational interventions.

*What is an educational intervention for facilitating foundation knowledge and skills in EBP?*

In this reporting guideline, an educational intervention is defined as ‘any learning activity where the intent is to facilitate the learning of skills or knowledge’. Evidence-based practice has been described as a ‘five-step model which forms the basis for clinical practice and teaching’ [2]. These five steps include ask, acquire, appraise, apply and assess [2]. Further detailed information for each of the five steps for EBP is provided on page 7. An educational intervention for EBP could be described by combining these two definitions, as an educational activity to improve skills and knowledge in EBP. Examples of educational interventions for EBP could include a conference workshop session on critical appraisal, an undergraduate learning session directed at asking a focussed clinical question and attendance at journal clubs and ward based case presentations. The items included in the GREET checklist are not recommendations for the best ways to teach or evaluate EBP education.

*The development of the GREET checklist*

The GREET checklist and the E&E paper have been developed using a transparent process [1,3]. Informed by a systematic review [3], a Delphi survey and expert consensus discussions, the inclusion of the checklist items aims to represent the minimum reporting requirements for an educational intervention for EBP. In the absence of empirical evidence, many of the GREET checklist items are based on consensus expert opinion. It is not intended to be a definitive list of reporting items.

The GREET checklist is based on the framework used for the Template for Intervention Description and Replication (TIDieR) checklist and guide, a generic checklist for reporting interventions [4]. TIDieR is intended to provide guidance for authors and editors for the reporting of interventions, for example a medication, surgery, physical therapy, where no specific guidance exists. The GREET checklist will provide reporting guidance for educational interventions that are specifically related to EBP.

*How to use the GREET checklist*

The GREET checklist includes items focussed on describing the intervention and does not include items concerning research design, or methodological issues such as recruitment, the psychometric properties of the outcome measures or the analysis approach. The GREET checklist is **not** intended to be used as a stand-alone document to cover all aspects of reporting an EBP educational study.

The GREET checklist and this E&E paper are intended to be used in conjunction with a reporting guideline specific to the study design. There are a variety of reporting guidelines for specific research and analysis approaches, many of which include at least one generic item for describing the intervention (Figure 1). Refer to the EQUATOR network (<http://www.equator-network.org/>) to find the most appropriate reporting guideline for a particular study design.

| **STEP 1:**  Select reporting guideline for **research approach** | |  | **STEP 2:**  Select reporting guideline for **intervention** |
| --- | --- | --- | --- |
| **EQUATOR network** (http://www.equator-network.org/). | |  |  |
| - Randomised controlled trials CONSORT[5] | |  | - Acupuncture STRICTA[6] |
| - SPIRIT Standard Protocol items for clinical trials [7] | |  | - Behaviour change WIDER Workgroup for Intervention Development and Evaluation Research [8] |
| - Observational studies STROBE [9] | |  | - Cancer pain educational interventions [10] |
| - Non- randomised designs TREND [11] | |  | - **Educational interventions for EBP (GREET)** [11] |
| - Interventions to improve quality and safety of care SQUIRE [12] | |  | - Generic reporting guidelines TIDieR [4] |
| - Case reports CARE [13] | |  | - Herbal interventions [14] |
| - Evaluations studies in health informatics STARE-HI [15] | |  | - Music-based interventions [16] |
|  |  | | - Non Pharmacological interventions [17] |
|  |  | | - Patient research reports in the medical education literature [18] |
|  |  | | - RedHOT homeopathic treatments [19] |
|  |  | | - Reporting the use of OSCEs [20] |
|  |  | | - Team Based learning [21] |

Figure 1 Steps in choosing an appropriate reporting guideline

The GREET checklist outlines the minimum requirements for reporting. Authors should provide further information they consider necessary, to enable the educational intervention to be replicated [4].

*When do you use the explanatory paper (E&E)?*

We recommend referring to this E&E paper whenever using the GREET checklist. This will help to report information with adequate detail. It may also assist to clarify any uncertainties or questions regarding the checklist items.

*How to use the E&E paper.*

The GREET checklist is provided on page 19. A brief explanation for each checklist item is provided in this E&E paper along with examples of reporting and clarification and troubleshooting suggestions for reporting complex interventions.

Each checklist item is independent. The items have been developed to ensure that each item can be understood without having to read the other checklist items.

*How to report an educational intervention for facilitating knowledge and skills in EBP.*

To limit the repetition in the instructions that follow, there a few things to note. Firstly and most importantly, it is critical that sufficient detail in the reporting is provided for the educational intervention in order for readers to consider how well the intervention might suit their own contexts and settings and potentially, to enable replication by someone else. This means that information should be reported for all groups involved in the educational intervention including the active and control or comparator groups. Describing the intervention for the control group as “usual care” is not sufficient. The same holds true for multicomponent interventions.

The order in which you report the checklist items is up to you and any specific journal requirements. Some of the checklist items may be able to be combined and incorporated into one sentence.

| **Explanation and elaboration for GREET checklist items** |
| --- |
| **1. INTERVENTION: Provide a brief description of the educational intervention for all groups involved [e.g. control and comparator(s)].** |
| The purpose of item 1 is to provide a brief, informative description of the educational intervention for each group(s) involved. Where appropriate, a one or two line description of the intervention delivery method (examples 1a-d), and EBP skills addressed (examples 1 c-e) is recommended. Inclusion of the name of the intervention in the abstract is recommended to enable the study to be easily identified as an educational intervention for EBP. Limit using abbreviations or acronyms. If they must be included, an explanation of the abbreviations and acronyms needs to be included (example 1b) [4]. |
| \| **Examples** \| \| \| --- \| --- \| \| **1a** \| “We conducted a cluster randomised controlled trial to compare a clinically integrated e-learning EBM course (intervention) to a lecture-based course (control) among post-graduate trainees at foundation or internship level in seven teaching hospitals in the UK West Midlands region.” [22] \| \| **1b** \| “A multifaceted evidence-based medicine (EBM) intervention was conducted at the largest health maintenance organization (HMO) in Israel, attempting to facilitate a change in doctors’ attitudes, knowledge and clinical behaviour.” [23] \| \| **1c** \| “An individual, 1-hour tutorial session conducted by health science librarians on the use of MEDLINE ….to improve MEDLINE utilization among residents training in obstetrics and gynaecology” [24] \| \| **1d** \| “The interventions consisted of (A) Computer based session and (B) Lecture based session. The content covered EBM teaching on question framing, literature searching, critical appraisal of systematic reviews and meta-analysis, and application of findings of systematic reviews, analogous to the problem-based approach in teaching and learning” [25] \| \| **1e** \| “We implemented a single, physician-led, hands-on 3h workshop to teach medical students clinical question formation and literature retrieval skills according to the first two steps of EBM…” [26] \| |
| What this item does not require: |
| It is not necessary to explain the entire intervention in the phrase for describing the intervention. A brief overview of the key elements of the study to enable easy identification of the type of intervention is ideal [4]. |
| Trouble shooting: |
| *What to do if there are multiple interventions?* |
| In studies where more than one educational intervention is employed, careful description in the intervention title is required to ensure that this information is reflected in the description of the intervention. Where possible it is recommended to include a brief description stating that more than one intervention has been employed, without providing the details of each intervention (example 1b). |
| **2. THEORY: Describe the educational theory (ies), concept or approach used in the educational intervention.** |
| Inclusion of the theory (ies) that underpin the intervention can provide a common language or foundation for describing the intervention [27]. This item requires a description of the theory (examples 2a-c), concept or approach (examples 2c,d) upon which the intervention was planned.  Describing or labelling the educational concept or theory used in the intervention can be difficult, as educational theories are often applied subconsciously, without identifying or labelling these educational processes as a specific theory, concept or approach [27]. Further information for educational theory and inter-professional education are provided in Kaufman (2003) [28] and Hean et al. (2012) [27]. |
| \| **Examples** \| \| \| --- \| --- \| \| ***Theory*** \| \| \| **2a** \| “The .. intervention was designed by the investigators based on the model of diffusion of innovation… and self-efficacy theory…” [29] \| \| **2b** \| “The directed students used a predetermined model for learning based on social learning theory.” [30] \| \| **2c** \| “This study was based upon Aizen’s Theory of Planned behaviour (TPB)…” [31] \| \| ***Concept or approach*** \| \| \| **2d** \| “..we undertook a ….trial with undergraduate medical students to test the effectiveness and learning satisfaction of …usual teaching versus problem based learning ..” [32] \| \| **2e** \| “Rogers’ model of innovation diffusion… was used to guide the organisation of different components of the intervention.” [33] \| |
| What this is item does not require: |
| Information regarding the learning objectives, intended outcomes or evaluation processes for the intervention does not actually describe the theoretical basis of the intervention. This information should be included in item 3, learning objectives, and the methods section as directed by the reporting guideline used in conjunction with the GREET checklist. |
| Trouble shooting |
| *What to do if there was no explicit theory on which the educational intervention was founded?* |
| Where an educational intervention has been designed without any underpinning theory, concept or approach, this should be reported. A statement such as, “*No specific educational theory was used in developing this educational intervention..*.” could be provided. |
| **3. LEARNING OBJECTIVES: Describe the learning objectives for all groups involved in the educational intervention.** |
| This item requires a description of the intended learning goals for all groups (intervention, comparator/s and/or control) in the educational intervention. Information should be provided regarding the specific knowledge (example 3a), skills (examples 3b,c), behaviours (example 3d) and attitudes (example 3d) that learners are expected to achieve as a result of participating in the educational intervention. An example of how learning objectives might be reported: “*By the end of this educational intervention the learners were expected to: (list objectives here).”* |
| \| **Examples** \| \| \| --- \| --- \| \| ***Knowledge*** \| \| \| **3a** \| ““…the objectives ..were to determine if a general medicine journal club…improves (1) reading habits, (2) objective **knowledge** of epidemiology and biostatistics, and (3) ability to utilize this knowledge in critically appraising the medical literature.” [34] \| \| ***Skills*** \| \| \| **3b** \| “The ….objectives were…(3) to help the participants assess their current **skills** in critically appraising the literature (4) teach critical appraisal principles..” [35] \| \| **3c** \| “The learning objectives …for both the stand-alone group and the e-learning group were the same….Upon completion of the course, participants should be competently able to:   - Generate structured questions arising from clinical problems in practice - Search relevant literature, identifying systematic reviews wherever possible - Assess the quality (validity) of systematic reviews and research included within them…” [22] \| \| ***Attitudes and behaviours*** \| \| \| **3d** \| “Through participation in this curriculum, they develop the skills, **behaviours** and **attitudes** required for this ‘evidence-based’ practice of medicine. These attributes are reflected in our learning objectives, listed in Table 2.” [36] \| |
| What this item does not require: |
| This item is not intended to include information about the intent of the instructors or their training and qualifications (this would be included in item 8 as appropriate). Information about the educational content (item 4), the delivery method (item 9) or schedule (item 11) of the educational intervention and the evaluation of the intervention is not included here. Information regarding the evaluation of the educational intervention should be included in the methods and results section as directed by the reporting guideline appropriate for the study design. These are not part of the GREET checklist. |

| Trouble shooting: | |
| --- | --- |
| *What to do if there were no specific learning objectives targeted for the educational intervention?* | |
| If the educational intervention was designed without any specific learning objectives this information should be reported. A statement such as, “*There were no specific learning objectives targeted in this educational intervention*”, could be used. | |
| **4. EBP CONTENT: List the foundation steps of EBP (ask, acquire, appraise, apply, assess) included in the educational intervention.** | |
| The foundation steps of EBP refers to the five step model described by Dawes et al. (2005) [2] (Table 1). | |
| \| **Table 1 Summary of the 5 steps of EBP [**2] \| \| \| \| --- \| --- \| --- \| \| **Step 1** \| Ask \| Translation of an uncertainty into an answerable question \| \| **Step 2** \| Acquire \| Systematic retrieval of best evidence available \| \| **Step 3** \| Appraise \| Critical appraisal of evidence for validity and clinical importance \| \| **Step 4** \| Apply \| Application of appraised evidence into practice.  Exploration of patient values and the acceptability of the answer. \| \| **Step 5** \| Assess \| Evaluation of performance \| | |
| A simple statement such as EBP was taught is not enough to determine the EBP content for the educational intervention. Looking at the EBP content of the intervention, consider which dimensions of the five step model described by Dawes et al. (2005) [2] you can identify in the intervention and describe these steps and how they were implemented. Authors can refer to the steps of EBP included in the educational content as numbers i.e. 1 to 5, or provide extra detail explaining each step (examples 4a-c). Further clarification about which steps of EBP were not included could be described. |  |
| \| **Examples** \| \| \| --- \| --- \| \| **4a** \| “The workshop…emphasized four steps in the EBM process a) developing an answerable question, b) finding the best available evidence, c) evaluation the evidence and d) applying the evidence to a patient care decision………… individually searching multiple evidence databases; evaluating individual articles for validity; calculating absolute risk reductions…and applying evidence to a patient care decision..” [37] \| \| **4b** \| “The EBM curriculum was based on published studies….the first session focussed on formulating clinical questions using the patient, intervention, comparison, outcome method. The remaining sessions focussed on therapy prevention, diagnosis and prognosis… Medical librarians introduced residents to electronic evidence-based synapses and summary resources including Ovid etc. ..” [38] \| \| **4c** \| “The core content of the course was based on the major steps in the evidence-based practice process, namely, how to ask an answerable clinical question; find evidence; critically appraise evidence for its validity, importance, and applicability; and integrate the evidence with clinical expertise and the patient’s values and circumstances…” [39] \| | |
| What this item does not require: | |
| This item does not include information regarding the learning objectives or goals for the intervention (item 3), the materials provided to the learners such as books or journal articles (item 5) or the delivery schedule (item 11) used for the intervention. This item does not relate to the evaluation of the learning of the EBP content, which should be included in the methods section as directed by the reporting guideline appropriate for the study design. | |
| Trouble shooting: | |
| The information regarding the EBP steps / content may be explicit within the learning objectives. If this is the case, you may choose not to repeat this information. | |

| **5 MATERIALS: Describe the specific educational materials used in the educational intervention. Include materials provided to the learners and those used in the training of educational intervention providers. Indicate where these can be accessed (e.g. online appendix, URL).** |  |
| --- | --- |
| The list of educational materials is comparable to the ‘ingredients’ required for a recipe [4]. A description of the educational materials made available to the learners in the educational intervention or used as part of the training for the instructors should be provided. Examples below include the educational materials used during the educational intervention (examples 5a, b). All materials provided during the training for the instructors of the intervention should be described. It may not be feasible to provide detailed information of the educational materials in the journal article or conference presentation, if this is the case, it would be useful to provide information of where these educational materials can be accessed (example 5c). | |
| \| **Examples** \| \| \| --- \| --- \| \| ***Materials provided to the learners as part of the educational intervention*** \| \| \| **5a** \| “..prior to participation in the conference, residents were provided one half-hour lecture about EBM. They were also provided a syllabus for independent study detailing the techniques of EBM, including pertinent *User’s Guide to the Medical Literature* from the EBM working group,” [40] \| \| **5b** \| “As materials for the course we use Sackett's 'blue book', and our EBM book in Spanish ..” [41] \| \| ***Details for where educational materials can be accessed*** \| \| \| **5c** \| “…online self-tutorial program to help healthcare professionals develop critical appraisal and information mastery skills. An overview of the program content (in French) can be accessed through the website <http://www.infocritique.fmed.ulaval.ca>...” [31] \| | |
| What this is item does not require: | |
| Descriptions regarding the instructor(s), setting, and method of delivery or schedule used for the educational intervention are not required here. This information is included in checklist items 8-12. | |
| Trouble shooting: | |
| Where the educational intervention is comprised of multiple interventions with different educational materials, information should be provided for each different component of the intervention. A summary table or grid could be used to illustrate this information (Table 2). | |
| **Table 2 Example of summary grid to display educational materials provided to the learner(s) in a** **multifaceted educational intervention** (Taylor et al. 2004) [42] | |
| \| **Intervention groups** \| **Stage(s) of intervention** \| \| \| \| \| \| --- \| --- \| --- \| --- \| --- \| --- \| \| **Stage 1** Pre-workshop \| \| **Stage 2** Workshop \| \| **Stage 3** Post workshop \| \| Control group \| Did not attend workshop or receive any materials \| Did not attend workshop or receive any materials \| \| Did not attend workshop or receive any materials \| \| \| Intervention group 1 \| - Workshop objectives. - Orientation guide. - Clinical scenario /questions - Systematic review paper. - Glossary. \| 3 hour workshop  Used materials from pre-workshop pack. \| \| - Introductory talk slides. - Systematic review checklist. - JAMA guidelines for systematic review \| \| | |

| **6. EDUCATIONAL STRATEGIES: Describe the teaching / learning strategies (e.g. tutorials, lectures, online modules) used in the educational intervention**. | |  |
| --- | --- | --- |
| Examples of teaching and learning strategies include tutorials (example 6a), lectures (example 6b), journal clubs (example 6c) and self-directed and online strategies (example 6d). Information for the teaching and learning strategies used for all groups in the educational intervention should be described. | |  |
| \| **Examples** \| \| \| --- \| --- \| \| **6a** \| “..the weekly clerkship tutorial.”[43] \| \| **6b** \| “…the clinically integrated e-learning course (intervention) and the traditional lecture based course (control)…”[44] \| \| **6c** \| “Intervention residents participated in a highly structured, monthly EBM journal club containing 3 primary elements. The first element was a case-based presentation format … The second element was structured worksheets…. The third element of the evidence-based journal club was close faculty supervision.”[45] \| \| **6d** \| “For those in the control group, the librarian observed the search, noting on the data collection form any independent knowledge of searching and any problem areas encountered. No feedback or instruction was provided…..For the test group participants, the librarian provided active instruction based on the nature of the question and the searcher’s level of skill.”[46] \| | |  |
| What this item does not require: | |  |
| This item does not include the learning theory upon which the educational strategies were based (item 2) or the materials provided to the learners as part of the teaching / learning strategies, this is included in materials (item 5). | |  |
| Trouble shooting: | |  |
| In the case where the educational intervention is comprised of more than one educational strategy and / or there are multiple learner groups involved, provide a summary for each of the different learning strategies for each component of the intervention. If the learning strategies were adapted for the learner(s) or group(s), further information should be provided in item 13 regarding the specific nature of the adaptation(s). | |  |
| **7. INCENTIVES: Describe any incentives or reimbursements provided to the learners.** | |  |
| An incentive is something that motivates an individual to perform an action [21] and could potentially act as a confounding variable. Incentives may be monetary or non-monetary. Examples of monetary incentives include honoraria to compensate for participants’ time (example 7a) and reimbursements for travel and parking costs, gift certificates / vouchers (example 7b). Whether learners paid a fee for the EBP education could be considered an incentive. Examples of non-monetary incentives include points accrued for professional continuing education programs (example 7c) and in some cases, assessment such as a compulsory examination or graded assignment may be considered an incentive. Information regarding any incentives or reimbursements provided to each learner(s) including when this was provided could be reported. | |  |
| \| **Examples** \| \| \| --- \| --- \| \| **7a** \| “A group of 22 trainees..volunteered to participate in the study. They were paid $100 for participating.”[47] \| \| **7b** \| “Provision of a coffee shop voucher encouraged participation in each focus group session..” [32] \| \| **7c** \| “No monetary compensation was offered, only CME accreditation.” [23] \| | |  |
| What this item does not require: | |  |
| This item is not whether or how much the instructors of the intervention were paid (item 5) or the venue cost for the course / workshop /seminar. This item does not include extensions of the study that ‘offer’ outreach support which is more specifically part of the study methodology [48]. | |  |
| Trouble shooting: | |  |
| *What to do if there were no incentives or reimbursements?* | |  |
| If no incentives or reimbursements were provided to participants, a simple statement such as “*No incentives / reimbursements were provided to participants”* could be reported. | |  |
| *What if there are multiple or different incentives or reimbursements?* | |  |
| For educational interventions that provide more than one incentive or reimbursement, information regarding each incentive or reimbursement that was provided to each group(s) or learner(s) could be specified. | |  |
| **8. INSTRUCTORS: For each instructor(s) involved in the educational intervention describe their professional discipline, teaching experience / expertise. Include any specific training related to the educational intervention provided for the instructor(s).** | |  |
| The professional discipline and teaching experience or expertise of the instructors of the educational intervention may potentially influence learning outcomes, yet information for the instructors of the educational intervention was amongst the least commonly reported information by studies of educational interventions in EBP [49]. Less than half of the studies in a recent systematic review reported information regarding the instructor’s profession (44%), the number of instructors involved (39%) or the instructor’s teaching experience or expertise (2%).  For each of the instructors involved in the educational intervention, describe their professional discipline (the broad profession of the instructor e.g. Epidemiologist, General Practitioner, Librarian or Physiotherapist) (example 8a) along with the instructor’s job title and any subspecialties where relevant. The teaching experience and expertise should be reported for each instructor and this may be described in a variety of ways, including the number of years experience of the instructors or the amount of time spent in specific educational or teaching positions facilitating the educational needs of learners (examples 8b,c). The instructor’s expertise such as special center / institute responsibilities could be provided as evidence of the instructor’s expertise. Any specific training requirements the instructors were required to undertake in order to be able to teach the educational intervention should be described (examples 8 d,e). | |  |
| \| **Examples** \| \| \| --- \| --- \| \| ***Profession / role*** \| \| \| **8a** \| “ Each tutorial was supervised jointly by a medical research librarian and 1-3 faculty members from the Division of General Internal Medicine.”[50] \| \| ***Teaching experience / expertise of the instructor(s)*** \| \| \| **8b** \| “The instructor … has 6 years’ experience in EBM training.” [26] \| \| **8c** \| “In the assisted group, participants were supported by a librarian with 5 years of experience….. (non- assisted group) was supported by.. two medical librarians with 1 year of experience ..” [51] \| \| ***Training provided to the instructors of the educational intervention*** \| \| \| **8d** \| “Three faculty members (instructors) trained in EBM through the annual McMaster University *How to teach evidence-based clinical practice workshop..”* [40] \| \| **8e** \| “Thirteen physicians (instructors) .. participated in a 12-hour pre-intervention EBM course...” [23] \| | |  |
| What this item does not require: | |  |
| This item does not include the materials that were provided to the instructors as part of the specific training, this information should be reported in item 5- Materials. This item does not include information regarding how (item 9 Delivery) or where (item 10 Environment) the instructors delivered the educational intervention(s) or to whom (number of learners – item 9). |  |  |
| Trouble shooting: | |  |
| *Is the instructor’s professional title the same thing as their professional discipline?* | |  |
| The professional discipline of the instructor(s) may not necessarily reflect the discipline for their professional role or job title. For example an academic employed within a medical training program may have a professional qualification in social work. In this case, the information reported regarding the professional discipline would be social work and the professional role for the instructor- lecturer in medicine should be reported. | |  |
| *What about complex interventions with multiple instructors?* | | |
| To simplify the description for complex educational interventions, such as those with multiple instructors with different professional backgrounds, expertise and training requirements a grid format may be used (Table 3). | | |

| **Table 3. Example table for an educational intervention with multiple instructors** (Gardois et al. 2011) [51]   \| **Learner groups** \| **Professional discipline** \| **Teaching experience/**  **expertise** \| **Specific training provided in order to be able to teach the intervention.** \| \| --- \| --- \| --- \| --- \| \| **Control group**  No of instructors= 2 \|  \|  \|  \| \| Instructor 1 \| Medical librarian \| ≥1yr \| Two medical librarians with ≥1 year of experience were selected and specifically trained … assigned to observe nine search sessions, recording in detail on a pre-structured form the actions performed by participants and assisting librarians during the accomplishment of the tasks. \| \| Instructor 2 \| Medical librarian \| ≥1yr \| Two medical librarians with ≥1 year of experience were selected and specifically trained … assigned to observe nine search sessions, recording in detail on a pre-structured form the actions performed by participants and assisting librarians during the accomplishment of the tasks. \| \| **Comparator group**  No of instructors=1 \|  \|  \|  \| \| Instructor 1 \| Medical librarian \| >5years \| None reported \| |
| --- | --- | --- | --- | --- | --- | --- | --- | --- | --- | --- | --- | --- | --- | --- | --- | --- | --- | --- | --- | --- | --- | --- | --- | --- |
| *What if information about the instructors was not collected?* |
| If there is limited or no information recorded regarding the instructor’s professional discipline, teaching experience /expertise or specific training requirements to teach the intervention, then this should be clearly stated. This could be done using the grid format above by inserting “not collected” into the appropriate column, or by using a statement such as “*Information regarding the instructors professional discipline / teaching experience or expertise / specific training provided in order to be able to teach the intervention, was not collected.”* |
| **9. DELIVERY: Describe the modes of delivery (e.g. face-to-face, internet or independent study package) of the educational intervention. Include whether the intervention was provided individually or in a group and the ratio of learners to instructors.** |
| The mode of delivery refers to the format in which the educational intervention was provided (e.g. face-to-face, online, journal club, telephone support, independent individually tailored remote study package). The numbers in the group(s) refers to whether the intervention was delivered to an individual, a group or class and the ratio of learners to instructors (e.g. 5:1, 3:1). To enable replication of the educational intervention, description of the mode(s) of delivery (example 9a) and whether the intervention was provided individually or in a group (example 9b) is required. The number of learners for each group should be reported with reference to the total number of instructors (examples 9a-c) and can be expressed as a ratio of instructors to learners (example 9d). |
| \| **Examples** \| \| \| \| \| --- \| --- \| --- \| --- \| \| ***Mode*** \| \| \| \| \| **9a** \| “In February 2006, the librarian conducted lecture / hands-on instruction for the entire ... 90 students .. The first 30-minute session introduced the students to CINAHL on the Ovid platform and showed them how to do a keyword search. The second session (carried out in groups of 15 students) included a 30-minute lecture/demonstration on how to conduct subject heading…. Searches followed by an hour of hands-on practice..” [52] \| \| \| \| **9b** \| “The e-learning materials were supplemented by two face-to-face group meetings to relieve the isolation that e-learners sometimes experience.” [53] \| \| \| \| ***Individual or group*** \| \| \| \| \| **9c** \| \| “The librarian sat with each resident individually in a private office in the NICU, while the resident attempted to find clinically relevant information in MEDLINE.”[46] \| \| \| ***Ratio of instructors to learners*** \| \| \| \| \| **9d** \| \| \| “Tutors and clerks in family medicine, medicine, pediatrics, and surgery clerkships at two of the four Hamilton, Ontario, teaching hospitals were allocated to the experimental intervention (11 tutors, 49 students), and those in these same rotations at the other two teaching hospitals acted as controls (11 tutors, 43 students).” [43] \| |

| What this item does not require: | |
| --- | --- |
| Information regarding where the educational intervention was delivered (item 10), or the materials used or provided to the learners (item 5) as part of the educational intervention is not required here. | |
| Trouble shooting: | |
| The information regarding the modes of delivery for the educational intervention may be explicit within the learning strategies. If this is the case, you may choose not to repeat this information. | |
| *What to do if there were multiple modes of delivery used in the educational intervention(s)?* | |
| In the situation where there are multiple interventions and multiple modes of delivery, this should be specified and the modes of delivery and learner / instructor ratio described for each group (s) in each part of the educational intervention (example 9a). | |
| *What to do if the number of learners / instructors was not reported?* | |
| If information regarding the number of learners / instructors in the educational intervention was not collected, this should be stated. A statement such as, “*The learner / instructor ratio was not able to be calculated as the total number of learners / instructors involved in the educational intervention was not recorded*” could be used. | |
| **10. ENVIRONMENT: Describe the relevant physical learning spaces (e.g. conference, university lecture theatre, hospital ward, community) where the teaching / learning occurred.** | |
| Details about the environment or physical learning space where the educational intervention took place may be relevant to the delivery, feasibility and generalisability of the intervention [4]. A brief description of the relevant settings where the educational intervention (s) took place should be reported (examples 10a-c). | |
| \| **Examples** \| \| \| --- \| --- \| \| **10a** \| “Residents … from two successive classes in the University of Wisconsin-Madison Internal Medicine Residency program in the treatment group participated in an interactive 4-hour EBM workshop …took place in a computer lab..” [37] \| \| **10b** \| “…two general units of a large Italian paediatric teaching hospital …both assisted and non-assisted search sessions were held in the library ….” [51] \| \| **10c** \| “Students randomized to the intervention participated in an EBM literature searching skills (EBM-LSS) workshop. The EBM-LSS workshop was two hours in duration and was delivered in the training room of the Hargrave-Andrew Library at the Clayton campus of Monash University.”[54] \| | |
| What this item does not require: | |
| A detailed description of the exact location, the size of the room (square feet) or contents of the physical learning space (e.g. number of chairs, computers) is not required. This is not a description of the location of employment or residence for the instructors. | |
| Trouble shooting: | |
| *How to report this for educational interventions with multiple parts and different settings?* | |
| For multi-faceted educational interventions where more than one physical learning space is used, describe all of the relevant physical learning space(s) used during the educational interventions for each of the learning groups. It is not unusual for part of an educational intervention to take place in a lecture room and another part in a library or computer pool. | |
| *How do you describe the learning environment for online interventions?* | |
| For educational interventions with an online component, the physical learning space would comprise the space where the access to the online resources where provided (e.g. learners home computer, library computer pool, hospital ward computer).  *What to do if this information was not recorded?* | |
| If the setting where the educational intervention was undertaken was not recorded this should be specified. A statement such as, “*The setting(s) for the educational intervention(s) was not recorded*” could be used. | |
| **11. SCHEDULE: Describe the scheduling of the educational intervention including the number of sessions, their frequency, timing and duration.** | |
| The schedule used for the educational intervention can be likened to describing the educational ‘dosage’ that was provided to the learners [55]. Detailed information regarding the schedule for an educational intervention is an essential requirement to enable replication, yet less than 70 per cent of studies in a recent systematic review reported the duration of the learning sessions for the educational intervention [49]. Specific details including the number, duration, and frequency of the learning sessions (examples 11a–d), the duration of the entire intervention program and spaces between learning sessions or facets of a multi-faceted intervention should be reported. The timing of the intervention, the stage of the year, location in the learners program as a whole (e.g. novice students or those about to graduate) and duration of the educational intervention, may potentially impact upon the learning outcomes. An example of this would be where an educational intervention spanned a large semester holiday break. Providing the start and finish dates for the educational intervention will enable the total duration and the timing of the intervention to be interpreted (examples 11a,b).  You will not necessarily need to report each of these items separately. It may be more suitable to combine some or all of the items to describe the scheduling for the educational intervention, as demonstrated in the examples below. | |
| \| **Examples** \| \| \| --- \| --- \| \| **11a** \| “…The study was carried out from September 2005 until the beginning of January 2006…the intervention group participated in an EBM training programme, consisting of an EBM course of three half-days spread over 2 weeks…paired with …approximately ten chairing case method learning sessions ….every other week and…lasted 1–1½ h.... for a period of 4 months….. .. the control group applied their usual standards of care during the 4-month intervention period..” [56] \| \| **11b** \| “The first cohort received the intervention in spring 2002 and the last in spring 2003…within the first 2 weeks of the 20-week tenth semester and consisted of 5 half-day sessions..” [30] \| \| **11c** \| “The experimental trial consisted of a weekly journal club, each seminar lasting 1.5 hours, for 12 weeks. …….The control group did not participate in a similar journal club during this period…” [57] \| \| **11d** \| “The course was run over a 13-week period (2 hours per week)..in the second semester of the academic year….” [39] \| | |
| What this item does not require: | |
| This item does not require a description of the learners involved in the educational intervention (e.g. number, professional discipline). Information for the learners is likely to be included in the results section as directed by the reporting guideline relevant for the study design you have used.  The evaluation of the delivery schedule and whether the educational intervention was delivered as scheduled is not required here. This information should be included in item 17.  It is possible that the educational schedule was not the same for all of the group(s) or learners in the educational intervention. If this is the case, the specific details regarding the adaptations that were made will be included in the next item (item 12 planned adaptations). Description of any prior EBP training or learning is not required for item 11. This may be useful information when describing the study participants, and may be included in the results section as directed by the reporting guideline you are using in conjunction with the GREET checklist. | |
| Trouble shooting: | |
| *What to do if information regarding part or all of the scheduling items was not collected?* | |
| If some or all of the scheduling information items were not collected this should be specified. For any /all of the scheduling information items that were not reported, a statement such as “*The total number of sessions / frequency / duration / timing of the educational sessions included in the educational intervention(s) were not recorded*” could be provided. |  |
| *What to do if there is more than one intervention with differing delivery schedules?* |  |
| In the situation where there are multiple interventions with different delivery schedules, this should be specified. The delivery schedule should be described for all of the learning sessions delivered to all of the learning groups in the educational intervention. | |

| **12. Describe the amount of time learners spent in face to face contact with instructors and any designated time spent in self-directed learning activities.** |
| --- |
| How much time the learners spent in designated learning activities may impact on their learning outcomes. Providing an indication of the total amount of learning time in face to face contact and self-directed learning may be useful. Only two per cent of studies in a recent systematic review reported the amount of time the learners spent in self-directed learning activities [49]. The amount of time the learner(s) spent in either face to face contact with the instructors or in designated self-directed learning activities (examples 12a,b) should be described. |
| \| **Examples** \| \| \| --- \| --- \| \| **12a** \| “Teaching is spread over four-half day sessions. Total contact time is 5 hours with 4.5 hours of directed self-study.” [58] \| \| **12b** \| “The EBM course consisted of 1-hour … conferences twice a week for 7 consecutive weeks and a 1.5-hour session in a computer lab…” [59] \| |
| What this item does not require: |
| This item does not require the amount of time the instructor(s) spent planning and/or preparing for the learning sessions. |
| Trouble shooting: |
| This information may have been covered if you have previously specified the amount of time spent in face to face contact and in self-directed learning activities in item 9 (delivery). You may choose not to repeat this information. |
| *What to do if this information was not collected?* |
| Where the details regarding the amount of time the learners spent in face to face contact with the instructors or in self-directed learning was not collected for the educational intervention, this information should be clearly stated. A statement such as, “*The amount of time the learners spent in (face to face contact and /or self-directed learning) was not recorded*” could be used. |
| **13. Did the educational intervention require specific adaptation for the learners? If yes, please describe the adaptations made for the learner(s) or group(s).** |
| Educational interventions are complex, and it is not always possible or appropriate for an educational intervention to follow a strict formula [60]. Educational interventions frequently require modification to ensure they meet the needs of the learner(s). This item describes any intentional adaptations that were made to the educational intervention to meet the specific learning needs of the individual(s) or group(s). These could include modifying educational materials from the original source (adding further information, modules, tasks etc.), altering the scheduling of the intervention to suit an available timeframe, changing scenarios to better reflect the professional disciplines of the learners, or converting an educational resource from its original medium to another medium (book/hard copy handouts to online materials).  The specific adaptations required to meet the learning needs of the individual(s) or groups(s) may include the language requirements for the learners (example 13a), substitution of examples in an assignment, one on one tutoring (example 13b) or assisted group work (example 13c) to meet the needs of each individual learner. |
| \| **Examples** \| \| \| --- \| --- \| \| **13a** \| “In order to avoid possible errors in the student’s understanding and assessment as a result of language, the articles selected were written in German..” [61] \| \| **13b** \| “We appointed a dedicated e-learning tutor to provide telephone and email tutorial support to the students….” [53] \| \| **13c** \| “All articles were in English and attendees with limited English proficiency were encouraged to work in groups” [62] \| |
| What this item does not require: |
| This item is not how closely the planned educational intervention matched the educational intervention that was actually delivered. This is sometimes referred to as the ‘fidelity’ of the intervention [63] and this information is covered in items 16 and 17. Unplanned or unintentional modifications that were made during the course of the educational interventions are included in item 14. |
| Troubleshooting: |
| *What about preserving the confidentially of the learner(s) or group(s)?* |
| In the case where describing the planned adaptations to the educational intervention may result in the loss of anonymity for the participant(s) or group(s) in the intervention, these planned modifications should be described broadly without providing identifying information. |
| *What to do if this information was not recorded?* |
| If no record of any adaptations was kept, then this should be clearly stated. A statement such as “*No record of any specific adaptations made to the educational intervention was kept*” could be used. |
| **14. Was the educational intervention modified during the course of the study? If yes, describe the changes (what, why, when, and how).** |
| This item describes any modifications that were made after the educational intervention had commenced which were not part of the original plan for the educational intervention. The detail(s) regarding what changes were required (example 14a), why they were made (examples 14a, b) and how (example 14c) and at what stage during the educational intervention (example14a) the change was made, should be described. |
| \| **Examples** \| \| \| --- \| --- \| \| **14a** \| “We made several changes in our resources … we converted access to all resources to the Internet via the public address, [http://Clinical.UTHSCSA.edu] . We replaced the Windows® inter- face on our clinical computers with a customized Internet browser whose home page was this Internet address. ..we created SUMSearch [http:// SUMSearch.UTHSCSA.edu] , an Internet site that automates searching for medical evidence. …we also changed our educational intervention. We reduced instruction on each individual resource, but we increased instruction on SUMSearch….” [64] \| \| **14b** \| “Based on the first performance assessment…the librarians and the nurse educator realized that 2 hours of instruction in a one-time session was not enough…. several interesting searching mistakes were revealed that guided our subsequent instruction….” [52] \| \| **14c** \| “In the first cohort, the University of Oslo inadvertently distributed the marking framework (intended for the assessors) to the students during the examination…” [30] \| |
| What this is item does not require: |
| This item does not include planned modifications made prior to commencing the educational intervention, such as translation of materials into another language or the provision of extra time for the individual learner(s). These planned modifications should be described in item 13. |
| Troubleshooting: |
| *What about multi-faceted interventions where multiple modifications were made?* |
| For multi-faceted educational interventions where more than one modification was made during the course of the educational intervention, you will need to describe each of the modifications for each of the learning groups. |
| *What to do if any modifications made during the course of the educational intervention were not recorded?* |
| If the details regarding any unplanned adaptations made during the course of the educational intervention were not recorded, this information should be clearly stated. A statement such as “*No record of any modifications made during the course of the educational intervention was retained*” could be used. |
| **15. ATTENDANCE: Describe the learner attendance, including how this was assessed and by whom. Describe any strategies that were used to facilitate attendance.** |
| The attendance of the learners, or lack thereof, in the educational intervention may impact on the learning outcomes [65]. Few studies to date have reported attendance, with only 39 per cent of studies reporting learner attendance in a recent systematic review [49]. Detailed information regarding learner attendance (example 15a), how the learner attendance was assessed (example 15b,c) and the person(s) involved in assessing the attendance should be described. Include information regarding any strategies that were undertaken to facilitate learner attendance for each group involved in the educational intervention (e.g. taking a roll, providing a mark for participation / attendance) (examples 15a-c). |
| \| **Examples** \| \| \| --- \| --- \| \| **15a** \| “Attendance rates were considered to provide indirect measures of intervention compliance for the directed learning group. However, this measure was inappropriate for the self-directed students, who were encouraged to choose the pace, time and site of their learning. ... Overall, 35% of self-directed and 73% of directed students attended at least 3 of 5 teaching sessions..” [30] \| \| **15b** \| “Attendance was kept and this was sent monthly to the research coordinator….In the moderated group, the mean resident attendance at each journal club ranged from 33% to 80.1%” [66] \| \| **15c** \| “Student participation was noted through small-group attendance and facilitator assessment of student preparation/participation…Student utilization of web-based supplemental curriculum was note through WebCT, which tracks log-in IDs per student, number of times pages are accessed (“hits”) and time spend per page”[67] \| |
| What this is not: |
| This item does not include a description of how well the actual educational intervention that was delivered matched the planned delivery or schedule for the educational intervention (item 17). Information regarding the assessment of learner dropouts or the evaluation of the learning outcomes is not required here. |
| Troubleshooting: |
| *What do if learner attendance was not assessed for the educational intervention?* |
| Where the details regarding the learner attendance for the educational intervention were not recorded this should be clearly stated. A statement such as “*Learner attendance for the educational intervention was not recorded*” could be used. |
| *What about complex interventions with multiple assessors and/ or assessment of learner attendance?* |
| Specific detail needs to be included in situations where there are multiple interventions and where learner attendance is assessed in different ways by different people. The learner attendance, including how this was assessed and who assessed attendance should be described for all of the learning sessions for each of the learning group(s) in the educational intervention. |
| *What about educational interventions that did not required physical attendance to the learning environment (e.g. self-directed or online learning, surveys, and assignments)?* |
| In the case of educational interventions that comprise self-directed learning, the reporting of attendance is not appropriate, though reporting the completion of the allocated task (assignment, survey) or log in rates / time spent in the site as a proxy may reflect the time learners spent completing the work (example 15a). |
| **16. Describe any processes used to determine whether the materials (item 5) and the educational strategies (item 6) used in the educational intervention were delivered as originally planned.** |
| The intervention-as-implemented in an experiment frequently differs from the intervention-as-designed [63]. The intention of this item is to describe any strategies or processes used to determine whether the planned teaching and learning strategies were delivered as originally intended. Examples of these processes include whether the session content and materials used in the educational intervention were observed (example 16a), videoed or checked (example 16b) to confirm they were delivered as planned or whether the website links were current. If there is a published study protocol for the educational intervention, provide reference to the where the study protocol can be accessed. |
| \| **Examples** \| \| \| --- \| --- \| \| **16a** \| “Two medical librarians with 1 year of experience were selected and specifically trained before the intervention; each one of them was then assigned to observe nine search sessions, recording in detail on a pre-structured form the actions performed by participants and assisting librarians during the accomplishment of the tasks. After the sessions, observers and researchers discussed the data emerging from the field, clarifying specific controversial points.” [51] \| \| **16b** \| “The implementation measure was derived from classroom observation ratings by trained project classroom observers… Observers blind to experimental condition …to evaluate the extent to which intervention and control teachers used the targeted teaching strategies. Each teacher was observed for four periods during the year..” [68] \| |
| What this item does not require: |
| The actual delivery schedule achieved for the educational intervention should be reported in item 17. Information regarding the assessment of the learning outcomes is not required here. Depending upon the journal requirements, the outcome of fidelity could be reported as part of the intervention quality check in the methods section or as part of the results. |
| Troubleshooting: |
| *What if no assessment was undertaken to determine whether the teaching and learning strategies used in the educational intervention were delivered as planned?* |
| If there were no evaluation processes to determine whether the materials and teaching / learning strategies used in the educational intervention were delivered as planned this should be clearly stated. A statement such as, “*No formal process was used to determine whether the materials and teaching / learning strategies used in the educational intervention were delivered as originally planned”* could be used*.* |
| **17. Describe the extent to which the number of sessions, their frequency, timing and duration for the educational intervention was delivered as scheduled (item 11).** |
| The extent to which the actual educational intervention was delivered as originally planned can impact on the success of the educational intervention (Nelson et al. 2012). This item describes how closely the actual delivery schedule of the educational intervention (the number of sessions, their frequency, timing and duration) matched the delivery schedule originally planned for the educational intervention (examples 17a-c). Information regarding whether the educational intervention was delivered as specified in the study protocol could be provided. |

|  |
| --- |
|  |
| \| **Examples** \| \| \| --- \| --- \| \| **17a** \| “The independent process evaluator observed two .. class-sessions per semester for each intervention school during spring semester of each intervention year…” [69] \| \| **17b** \| “.. all teachers were scheduled to be observed at least once during the 6 week curriculum implementation cycle. Teachers were informed about the observation during the training and contacted a few days prior to schedule their observation. Raters were four doctoral or masters level students, trained by senior staff. Using a standardized form, observers rated (scored yes/no) whether teachers implemented each of the procedures contained in the lesson..” [70] \| \| **17c** \| “To obtain a measure of program dosage, teachers were asked to report the number of lessons that they taught each week. In addition, program fidelity was assessed by the project staff with direct observations of the teachers in the context of their ongoing consultation…” [55] \| |
| What this item does not require: |
| This is not how well the learning objectives were met or the learning outcomes achieved. |
| Troubleshooting: |
| If the modifications made to the delivery schedule for the educational intervention have been previously specified in items 13 and 14, you may choose not to repeat this information. |
| *What if no assessment was undertaken to determine the extent to which the number of sessions, their frequency, timing and duration for the educational intervention were delivered as scheduled?* |
| If there were no evaluation processes undertaken to determine the extent to which the number of sessions, their frequency, timing and duration for the educational intervention were delivered as scheduled this should be clearly stated. A statement such as, “*No formal assessment of the delivery schedule for the educational intervention was undertaken. Whether the number of sessions, their frequency, timing and duration were delivered as originally scheduled is not able to be determined for this educational intervention*” could be used. |

| **Conclusion** | |
| --- | --- |
| The GREET checklist is intended to provide a guide for authors and researchers when reporting educational interventions for EBP. The E&E paper has been developed to make the GREET checklist easier to use and understand. Ideally the aim of the GREET checklist is to enable more consistent and transparent reporting for educational interventions for EBP. Improving the quality of reporting for educational interventions for EBP could potentially benefit educators, researchers, journal editors and the learners in EBP education.  The next step for the GREET checklist and the E&E paper requires dissemination to ensure the uptake of these documents [1]. We have started spreading the word. The GREET checklist is registered on the equator website (<http://www.equator-network.org/>) and aligns with the TIDieR guidance [4].You can help with the dissemination process by spreading the word about the importance of transparent and complete reporting for educational interventions in EBP, by using the GREET checklist yourself and recommending it to your colleagues.  The GREET checklist and the E&E paper are intended to be evolving documents. We would value your feedback regarding the GREET checklist and the E&E paper. Please provide any feedback directly to Dr Anna Phillips, [anna.phillips@unisa.edu.au](mailto:anna.phillips@unisa.edu.au). We plan to update these documents in the future as more evidence emerges. |  |

| **GREET 2015 checklist*** based upon the TIDieR guidance |  |
| --- | --- |
| BRIEF NAME | |
| 1. INTERVENTION: Provide a brief description of the educational intervention for all groups involved [e.g. control and comparator(s)]. | |
| WHY - this educational process | |
| 2. THEORY: Describe the educational theory (ies), concept or approach used in the intervention. | |
| 3. LEARNING OBJECTIVES: Describe the learning objectives for all groups involved in the educational intervention. | |
| 4. EBP CONTENT: List the foundation steps of EBP (ask, acquire, appraise, apply, assess) included in the educational intervention. | |
| WHAT | |
| 5. MATERIALS: Describe the specific educational materials used in the educational intervention.  Include materials provided to the learners and those used in the training of educational intervention providers. | |
| 6. EDUCATIONAL STRATEGIES: Describe the teaching / learning strategies (e.g. tutorials, lectures, online modules) used in the educational intervention. | |
| 7. INCENTIVES: Describe any incentives or reimbursements provided to the learners. | |
| WHO PROVIDED | |
| 8. INSTRUCTORS: For each instructor(s) involved in the educational intervention describe their professional discipline, teaching experience / expertise. Include any specific training related to the educational intervention provided for the instructor(s). | |
| HOW | |
| 9. DELIVERY: Describe the modes of delivery (e.g. face-to-face, internet or independent study package) of the educational intervention. Include whether the intervention was provided individually or in a group and the ratio of learners to instructors. | |
| WHERE | |
| 10. ENVIRONMENT: Describe the relevant physical learning spaces (e.g. conference, university lecture theatre, hospital ward, community) where the teaching / learning occurred. | |
| WHEN and HOW MUCH | |
| 11. SCHEDULE: Describe the scheduling of the educational intervention including the number of sessions, their frequency, timing and duration. | |
| 12. Describe the amount of time learners spent in face to face contact with instructors and any designated time spent in self-directed learning activities. | |
| PLANNED CHANGES | |
| 13. Did the educational intervention require specific adaptation for the learners? If yes, please describe the adaptations made for the learner(s) or group(s). | |
| UNPLANNED CHANGES | |
| 14. Was the educational intervention modified **during** the course of the study? If yes, describe the changes (what, why, when, and how). | |
| HOW WELL | |
| 15. ATTENDANCE: Describe the learner attendance, including how this was assessed and by whom. Describe any strategies that were used to facilitate attendance. | |
| 16. Describe any processes used to determine whether the materials (item 5) and the educational strategies (item 6) used in the educational intervention were delivered as originally planned**.** | |
| 17. Describe the extent to which the number of sessions, their frequency, timing and duration for the educational intervention was delivered as scheduled (item 11). | |

***based on the TIDieR guidance. We strongly recommend reading this statement in conjunction with the GREET 2015 explanation and elaboration paper for important clarifications on all the items. If relevant, we also recommend reading the TIDieR guidance (Hoffman et al. 2014)**

**Reference list**

1. Moher D, Schulz KF, Simera I, Altman DG: Guidance for developers of health research reporting guidelines. PLoS Med 2010,7(2):e1000217.

2. Dawes M, Summerskill W, Glasziou P, Cartabellotta A, Martin J, Hopayian K, Porszolt F, Burls A, Osborne J and Second International Conference of Evidence-Based Health Care Teachers and Developers 2005: Sicily statement on evidence-based practice. BMC Med Educ 2005, 5(1):1.

3. Phillips AC, Lewis LK, McEvoy MP, Galipeau J, Glasziou P, Hammick M, Moher D, Tilson JK, Williams MT: Protocol for development of the guideline for reporting evidence based practice educational interventions and teaching (GREET) statement. BMC Med Educ 2013,13:9.

4. Hoffmann TC, Glasziou PP, Boutron I, Milne R, Perera R, Moher D, Altman DG, Barbour V, Macdonald H, Johnston M, Lamb SE, Dixon-Woods M, McCulloch P, Wyatt JC, Chan A, Michie S: Better reporting of interventions: Template for intervention description and replication (TIDieR) checklist and guide. BMJ 2014, 348.

5. Schulz KF, Altman DG, Moher D, CONSORT Group: CONSORT 2010 statement: Updated guidelines for reporting parallel group randomised trials. BMC Med 2010, 8:18.

6. MacPherson H, Altman DG, Hammerschlag R, Youping L, Taixiang W, White A, Moher D and STRICTA Revision Group: Revised STandards for reporting interventions in clinical trials of acupuncture (STRICTA): Extending the CONSORT statement. PLoS Med 2010,7(6):e1000261.

7. Chan AW, Tetzlaff JM, Altman DG, Laupacis A, GotzschePC, Krleza-Jeric K, Hrpbjartsson A, Mann H, Disckersin K, Berlin JA, Dore CJ, Parulekar WR, Summerskill WS, Groves T, Schulz KF, Sox HC, Rockhold FW, Rennie D, Moher D: SPIRIT 2013 statement: Defining standard protocol items for clinical trials. Ann Intern Med 2013, 158(3):200-207.

8. Albrecht L, Archibald M, Arseneau D, Scott SD: Development of a checklist to assess the quality of reporting of knowledge translation interventions using the workgroup for intervention development and evaluation research (WIDER) recommendations. Implement Sci 2013, 8:52-5908-8-52.

9. von Elm E, Altman DG, Egger M, Pocock SJ, Gøtzsche PC, Vandenbroucke JP: The strengthening the reporting of observational studies in epidemiology (STROBE) statement: Guidelines for reporting observational studies. Epidemiology 2007,18(6):800-804.

10. Stiles CR, Biondo PD, Cummings G, Hagen NA. Clinical trials focusing on cancer pain educational interventions: Core components to include during planning and reporting. J Pain Symptom Manage 2010, 40(2):301-308.

11. Des Jarlais DC, Lyles C, Crepaz N, TREND Group: Improving the reporting quality of nonrandomized evaluations of behavioral and public health interventions: The TREND statement. Am J Public Health 2004, 94(3):361-366.

12. Davidoff F, Batalden P, Stevens D, Ogrinc G, Mooney SE, SQUIRE development group: Publication guidelines for quality improvement studies in health care: Evolution of the SQUIRE project. BMJ. 2009, 338:a3152.

13. Gagnier JJ, Kienle G, Altman DG, Moher D, Sox H, Riley D; the CARE Group: The CARE guidelines: Consensus-based clinical case report guideline development. J Diet Suppl 2013, 10(4):381-390.

14. Gagnier JJ, Boon H, Rochon P, Moher D, Barnes J, Bombardier C: Reporting randomized, controlled trials of herbal interventions: An elaborated CONSORT statement. Ann Intern Med 2006, 144(5):364-367.

15. Talmon J, Ammenwerth E, Brender J, de Keizer N, Nykanen P, Rigby M: STARE-HI-statement on reporting of evaluation studies in health informatics. Int J Med Inform 2009, 78(1):1-9.

16. Robb SL, Burns DS, Carpenter JS:Reporting guidelines for music-based interventions. J Health Psychol. 2011, 16(2):342-352.

17. Boutron I, Moher D, Altman DG, Schulz KF, Ravaud P, CONSORT Group: Extending the CONSORT statement to randomized trials of nonpharmacologic treatment: Explanation and elaboration. Ann Intern Med 2008,148(4):295-309.

18. Howley L, Szauter K, Perkowski L, Clifton M, McNaughton N, Association of Standardized Patient Educators (ASPE): Quality of standardised patient research reports in the medical education literature: Review and recommendations. Med Educ 2008,42(4):350-358.

19. Dean ME, Coulter MK, Fisher P, Jobst K, Walach H, Delphi Panel of the CONSORT Group: Reporting data on homeopathic treatments (RedHot): A supplement to CONSORT*. Forsch Komplementmed. 2006, 13(6):368-371.

20. Patricio M, Juliao M, Fareleira F, Young M, Norman G, Vaz Carneiro A: A comprehensive checklist for reporting the use of OSCEs. Med Teach 2009, 31(2):112-124.

21. Haidet P, Levine RE, Parmelee DX, Crow S, Kennedy F, Kelly PA, Perkowski L, Michaelsen L, Richards BF: Perspective: Guidelines for reporting team-based learning activities in the medical and health sciences education literature. Acad Med 2012, 87(3):292-299.

22. Hadley J, Kulier R, Zamora J, Coppus S, Weinbrenner S, Meyerrose B, Decsi T, Horvath AR, Nagy E, Emparanza JI, Arvanitis TN, Burls A, Cabello JB, Kaczor M, Zanrei G, Pierer K, Kunz R, Wilkie V, Wall D, Mol BWJ, Khan KS: Effectiveness of an e-learning course in evidence-based medicine for foundation (internship) training. Journal of the Royal Society of Medicine 2010,103(7):288-294.

23. Shuval K, Berkovits E, Netzer D, Hekselman I, Linn S, Brezis M, Reis S: Evaluating the impact of an evidence-based medicine educational intervention on primary care doctors' attitudes, knowledge and clinical behaviour: A controlled trial and before and after study. J Eval Clin Pract 2007, 13(4):581-598.

24. Erickson S, Warner ER: The impact of an individual tutorial session on MEDLINE use among obstetrics and gynaecology residents in an academic training programme: A randomized trial. Med Educ1998, 32(3):269-273.

25. Davis J, Crabb S, Rogers E, Zamora J, Khan K: Computer-based teaching is as good as face to face lecture-based teaching of evidence based medicine: A randomized controlled trial. Med Teach 2008, 30(3):302-307.

26. Sastre EA, Denny JC, McCoy JA, McCoy AB, Spickard A: Teaching evidence-based medicine: Impact on students' literature use and inpatient clinical documentation. Med Teach 2011, 33(6):e306-12.

27. Hean S, Craddock D, Hammick M, Hammick M: Theoretical insights into interprofessional education: AMEE guide no. 62. Med Teach 2012, 34(2):e78-101.

28. Kaufman DM: Applying educational theory in practice. BMJ 2003, 326(7382):213-216.

29. Kim SC, Brown CE, Fields W, Stichler JF: Evidence-based practice-focused interactive teaching strategy: A controlled study. J Adv Nurs 2009, 65(6):1218-1227.

30. Bradley P, Oterholt C, Herrin J, Nordheim L, Bjorndal A: Comparison of directed and self-directed learning in evidence-based medicine: A randomised controlled trial. Med Educ 2005, 39(10):1027-1035.

31. Gagnon MP, Legare F, Labrecque M, Fremont P, Cauchon M, Desmartis M: Perceived barriers to completing an e-learning program on evidence-based medicine. Informatics in Primary Care 2007, 15(2):83-91.

32. Johnston JM, Schooling CM, Leung GM: A randomised-controlled trial of two educational modes for undergraduate evidence-based medicine learning in asia. BMC Med Educ 2009,9.

33. Forsetlund L, Bradley P, Forsen L, Nordheim L, Jamtvedt G, Bjorndal A: Randomised controlled trial of a theoretically grounded tailored intervention to diffuse evidence-based public health practice [ISRCTN23257060]. BMC Med Educ 2003, 3:2.

34. Linzer M, Brown J, Frazier L, DeLong E, Siegel W: Impact of a medical journal club on house-staff reading habits, knowledge, and critical-appraisal skills - a randomized control trial. JAMA 1988, 260(17):2537-2541.

35. Seelig CB: Changes over time in the knowledge acquisition practices of internists. South Med J. 1993, 86(7):780-783.

36. Green ML, Ellis PJ: Impact of an evidence-based medicine curriculum based on adult learning theory. Journal of General Internal Medicine 1997,12(12):742-750.

37. Feldstein DA, Maenner MJ, Srisurichan R, Roach MA, Vogelman BS: Evidence-based medicine training during residency: A randomized controlled trial of efficacy. BMC Med Educ 2010, 10:59.

38. Kim S, Willett LR, Murphy DJ, O'Rourke K, Sharma R, Shea JA: Impact of an evidence-based medicine curriculum on resident use of electronic resources: A randomized controlled study. JGIM: Journal of General Internal Medicine 2008, 23(11):1804-1808.

39. Bennett S, Hoffmann T, Arkins M: A multi-professional evidence-based practice course improved allied health students' confidence and knowledge. J Eval Clin Pract 2011, 17(4):635-639.

40. Thomas KG, Thomas MR, York EB, Dupras DM, Schultz HJ, Kolars JC:Teaching evidence-based medicine to internal medicine residents: The efficacy of conferences versus small-group discussion. Teach Learn Med 2005,17(2):130-135.

41. Sanchez-Mendiola M, Kieffer-Escobar LF, Marin-Beltran S, Downing SM, Schwartz A:Teaching of evidence-based medicine to medical students in mexico: A randomized controlled trial. BMC Med Educ 2012, 12:107.

42. Taylor RS, Reeves BC, Ewings PE, Taylor RJ: Critical appraisal skills training for health care professionals: A randomized controlled trial [ISRCTN46272378]. BMC Med Educ 2004, 4(1):30.

43. Bennett KJ, Sackett DL, Haynes RB, Neufeld VR, Tugwell P, Roberts R: A controlled trial of teaching critical appraisal of the clinical literature to medical students. JAMA 1987, 257(18):2451-2454.

44. Kulier R, Coppus S, Zamora J, Hadley J, Malick S, Das K, Weinbrenner B, Decsi T, Horvath AR, Nagy E, Emparanza JI, Arvanitis TN, Burls A, Cabello JB, Kaczor M, Zanrei G, Pierer K, Stawiarz K, Kinz R, Mp; BWJ, Khan KS: The effectiveness of a clinically integrated e-learning course in evidence-based medicine: A cluster randomised controlled trial. BMC Med Educ 2009;9.

45. Bazarian J, Davis C, Spillane L, Blumstein H, Schneider S: Teaching emergency medicine residents evidence-based critical appraisal skills: A controlled trial. Ann Emerg Med 1999, 34(2):148-154.

46. Bradley DR, Rana GK, Martin PW, Schumacher RE: Real-time, evidence-based medicine instruction: A randomized controlled trial in a neonatal intensive care unit. J Med Libr Assoc 2002, 90(2):194-201.

47. Falzer PR, Garman DM: Evidence-based decision-making as a practice-based learning skill: A pilot study. Academic Psychiatry 2012, 36(2):104-109.

48. McCluskey A, Lovarini M: Providing education on evidence-based practice improved knowledge but did not change behaviour: A before and after study. BMC Med Educ 2005, 5:40.

49. Phillips AC, Lewis LK, McEvoy MP, Galipeau J, Glasziou P, Hammick M, Moher D, Tilson JK, Williams MT: A systematic review of how studies describe educational interventions for evidence-based practice: Stage 1 of the development of a reporting guideline. BMC Med Educ 2014,14:152.

50. Stark R, Helenius IM, Schimming LM, Takahara N, Kronish I, Korenstein D: Real-time EBM: From bed board to keyboard and back. Journal of General Internal Medicine 2007, 22(12):1656-1660.

51. Gardois P, Calabrese R, Colombi N, Deplano A, Lingua C, Longo F, Villanacci MC, Piga A: Effectiveness of bibliographic searches performed by paediatric residents and interns assisted by librarians. A randomised controlled trial. Health Information and Libraries Journal 2011, 28(4):273-284.

52. Carlock D, Anderson J: Teaching and assessing the database searching skills of student nurses. Nurse Educ 2007, 32(6):251-255.

53. Webber M, Currin L, Groves N, Hay D, Fernando N: Social workers can e-learn: Evaluation of a pilot post-qualifying e-learning course in research methods and critical appraisal skills for social workers. Social Work Education 2010, 29(1):48-66.

54. Ilic D, Tepper K, Misso M: Teaching evidence-based medicine literature searching skills to medical students during the clinical years: A randomized controlled trial. Journal of the Medical Library Association 2012, 100(3):190-196.

55. Domitrovich CE, Greenberg MT: The study of implementation: Current findings from effective programs that prevent mental disorders in school-aged children. Journal of Educational and Psychological Consultation 2000,11(2):193-221.

56. Hugenholtz NIR, Schaafsma FG, Nieuwenhuijsen K, van Dijk FJH: Effect of an EBM course in combination with case method learning sessions: An RCT on professional performance, job satisfaction, and self-efficacy of occupational physicians. Int Arch Occup Environ Health 2008,82(1):107-115.

57. Fu C, Hodges B, Regehr G, Goldbloom D, Garfinkel P: Is a journal club effective for teaching critical appraisal skills? A controlled trial with residents in psychiatry. Academic Psychiatry 1999,23:205-209.

58. Edwards R, White M, Gray J, Fischbacher C: Use of a journal club and letter-writing exercise to teach critical appraisal to medical undergraduates. Med Educ 2001, 35(7):691-694.

59. Smith W: Evidence for the effectiveness of techniques to change physician behavior. Chest 2000,118(2):8S-17S.

60. Olson CA, Bakken LL: Evaluations of educational interventions: Getting them published and increasing their impact. J Contin Educ Health Prof 2013,33(2):77-80.

61. Arlt SP, Heuwieser W: Training students to appraise the quality of scientific literature. Journal of Veterinary Medical Education 2011,38(2):135-140.

62. Tomatis C, Taramona C, Rizo-Patron E, Hernandez F, Rodriguez P, Piscoya A, Gonzales E, Gotuzzo E, Heudebert G, Centor RM, Estrada CA: Evidence-based medicine training in a resource-poor country, the importance of leveraging personal and institutional relationships. J Eval Clin Pract 2011, 17(4):644-650.

63. Nelson MC, Cordray DS, Hulleman CS, Darrow CL, Sommer EC: A procedure for assessing intervention fidelity in experiments testing educational and behavioral interventions. J Behav Health Serv Res 2012, 39(4):374-396.

64. Badgett RG, Paukert JL, Levy LS: Teaching clinical informatics to third-year medical students: Negative results from two controlled trials. BMC Med Educ 2001,1:3.

65. Cheng GY: Educational workshop improved information-seeking skills, knowledge, attitudes and the search outcome of hospital clinicians: A randomised controlled trial. Health Info Libr J 2003, 20 Suppl 1:22-33.

66. McLeod RS, MacRae HM, McKenzie ME, Victor JC, Brasel KJ: Evidence Based Reviews in Surgery Steering Committee. A moderated journal club is more effective than an internet journal club in teaching critical appraisal skills: Results of a multicenter randomized controlled trial. J Am Coll Surg 2010, 211(6):769-776.

67. Srinivasan M, Weiner M, Breitfeld PP, Brahmi F, Dickerson KL, Weiner G: Early introduction of an evidence-based medicine course to preclinical medical students. Journal of General Internal Medicine 2002,17(1):58-65.

68. Abbott RD, O'Donnell J, Hawkins JD, Hill KG, Kosterman R, Catalano RF: Changing teaching practices to promote achieving and bonding to school. Am J Orthopsychiatry 1998, 68(4):542-552.

69. Saunders RP, Ward D, Felton GM, Dowda M, Pate RR. Examining the link between program implementation and behavior outcomes in the lifestyle education for activity program (LEAP). Eval Program Plann. 2006, 29(4):352-364.

70. Resnicow K, Davis M, Smith M, Lararus-Yaroch A, Baranowski T, Baranowski J: How best to measure implementation of school health curricula: A comparison of three measures. Health Educ Res 1998, 13(2):239-250.

**List of references used for the examples in the E&E paper**

| 1a | Hadley J, Kulier R, Zamora J, Coppus S, Weinbrenner S, Meyerrose B, Khan K S: Effectiveness of an e-learning course in evidence-based medicine for foundation (internship) training. Journal of the Royal Society of Medicine 2010, 103(7):288-294. |
| --- | --- |
| 1b | Shuval K, Berkovits E, Netzer D, Hekselman I, Linn S, Brezis M, Reis S: Evaluating the impact of an evidence-based medicine educational intervention on primary care doctors' attitudes, knowledge and clinical behaviour: A controlled trial and before and after study. Journal of Evaluation in Clinical Practice 2007, 13(4):581-598. |
| 1c | Erickson S, Warner E R: The impact of an individual tutorial session on MEDLINE use among obstetrics and gynaecology residents in an academic training programme: A randomized trial. Medical Education 1998, 32(3): 269-273. |
| 1d | Davis J, Crabb S, Rogers E, Zamora J, Khan K: Computer-based teaching is as good as face to face lecture-based teaching of evidence based medicine: A randomized controlled trial. Medical Teacher 2008, 30(3): 302-307. |
| 1e | Sastre EA, Denn JC, McCoy J, McCoy AB, Spickard A: Teaching evidence-based medicine: Impact on students’ literature use and inpatient clinical documentation. Medical Teacher 2011, 33(6): e306-12. |
| 2a | Kim SC, Brown CE, Fields W Stichler JF: Evidence-based practice-focused interactive teaching strategy: A controlled study. Journal of Advanced Nursing 2009, 65(6): 1218-1227. |
| 2b | Bradley P, Oterholt C, Herrin J, Nordheim L, Bjorndal A: Comparison of directed and self-directed learning in evidence-based medicine: A randomised controlled trial. Med Educ 2005, 39:1027-1035. |
| 2c | Gagnon MP, Legare F, Labrecque M, Fremont P, Cauchon M, Desmartis M: Perceived barriers to completing an e-learning program on evidence-based medicine. Informatics in Primary Care 2007, 15:83-91. |
| 2d | Johnston JM, Schooling CM, Leung GM: A randomised-controlled trial of two educational modes for undergraduate evidence-based medicine learning in Asia. BMC Med Educ 2009, 9:63 |
| 2e | Forsetlund L, Bradley P, Forsen L, Nordheim L, Jamtvedt G, Bjorndal A: Randomised controlled trial of a theoretically grounded tailored intervention to diffuse evidence-based public health practice [ISRCTN23257060]. BMC Med Educ 2003, 3:2. |
| 3a | Linzer M, Brown J, Frazier L, Delong E, Siegel W: Impact of a medical journal club on house-staff reading habits, knowledge, and critical-appraisal skills - a randomized control trial. J Am Med Assoc 1988, 260:2537-2541. |
| 3b | Seelig CB: Changes over time in the knowledge acquisition practices of internists. South Med J 1993, 86:780-783. |
| 3c | Hadley J, Kulier R, Zamora J, Coppus S, Weinbrenner S, Meyerrose B, Khan K S: Effectiveness of an e-learning course in evidence-based medicine for foundation (internship) training. Journal of the Royal Society of Medicine 2010, 103(7):288-294. |
| 3d | Green ML, Ellis PJ: Impact of an evidence-based medicine curriculum based on adult learning theory. Journal of General Internal Medicine 1997, 12:742-750. |
| 4a | Feldstein DA, Maenner MJ, Srisurichan R, Roach MA, Vogelman BS: Evidence-based medicine training during residency: A randomized controlled trial of efficacy. BMC Med Educ 2010, 10:59. |
| 4b | Kim S, Willett LR, Murphy DJ, O'Rourke K, Sharma R, Shea JA: Impact of an evidence-based medicine curriculum on resident use of electronic resources: A randomized controlled study. Journal of General Internal Medicine 2008, 23:1804-1808. |
| 4c | Bennett S, Hoffmann T, Arkins M: A multi-professional evidence-based practice course improved allied health students’ confidence and knowledge. J Eval Clin Pract 2011, 17(4): 635-639. |
| 5a | Thomas KG, Thomas MR, York EB, Dupras DM, Schultz HJ, Kolars JC: Teaching evidence-based medicine to internal medicine residents: The efficacy of conferences versus small-group discussion. Teach Learn Med 2005, 17:130-135. |
| 5b | Sánchez-Mendiola M: Evidence-based medicine teaching in the mexican army medical school. Med Teach 2004, 26:661-663. |
| 5c | Gagnon MP, Legare F, Labrecque M, Fremont P, Cauchon M, Desmartis M: Perceived barriers to completing an e-learning program on evidence-based medicine. Informatics in Primary Care 2007, 15:83-91. |
| 6a | Bennett KJ, Sackett DL, Haynes RB, Neufeld VR, Tugwell P, Roberts R: A controlled trial of teaching critical appraisal of the clinical literature to medical students. J Am Med Asscoc 1987, 257:2451-2454. |
| 6b | Kulier R, Coppus S, Zamora J, Hadley J, Malick S, Das K, Weinbrenner S, Meyerrose B, Decsi T, Horvath AR, Nagy E, Emparanza JI, Arvanitis TN, Burls A, Cabello J, Kaczor M, Zanrei G, Peirer K, Stawiarz K, Kunz R, Mol B, Khan KS: The effectiveness of a clinically integrated e-learning course in evidence-based medicine: A cluster randomised controlled trial. BMC Med Educ 2009, 9:21. |
| 6c | Bazarian J, Davis C, Spillane L, Blumstein H, Schneider S: Teaching emergency medicine residents evidence-based critical appraisal skills: A controlled trial. Ann Emerg Med 1999, 34:148-154. |
| 6d | Bradley DR, Rana GK, Martin PW, Schumacher RE: Real-time, evidence-based medicine instruction: A randomized controlled trial in a neonatal intensive care unit. J Med Libr Assoc 2002, 90:194-201. |
| 7a | Falzer PR, Garman DM: Evidence-based decision making as a practice-based learning skill: a pilot study. Academic Psychiatry 2012, 36(2):104-109. |
| 7b | Johnston JM, Schooling CM, Leung GM: A randomised-controlled trial of two educational modes for undergraduate evidence-based medicine learning in Asia. BMC Medical Educ 2009, 9:63 |
| 7c | Shuval K, Berkovits E, Netzer D, Hekselman I, Linn S, Brezis M, Reis S: Evaluating the impact of an evidence-based medicine educational intervention on primary care doctors' attitudes, knowledge and clinical behaviour: A controlled trial and before and after study. Journal of Evaluation in Clinical Practice 2007, 13(4):581-598. |
| 8a | Stark R, Helenius IM, Schimming LM, Takahara N, Kronish I, Korenstein D: Real-time EBM: From bedboard to keyboard and back. Journal of General Internal Medicine 2007, 22:1656-1660. |
| 8b | Sastre EA, Denn JC, McCoy J, McCoy AB, Spickard A: Teaching evidence-based medicine: Impact on students’ literature use and inpatient clinical documentation. Medical Teacher 2011, 33(6): e306-12. |
| 8c | Gardois P, Calabrese R, Colombi N, Deplano A, Lingua C, Longo F, Villanacci MC, Miniero R, Piga A: Effectiveness of bibliographic searches performed by paediatric residents and interns assisted by librarians. A randomised controlled trial. Health Information and Libraries Journal 2011, 28:273-284. |
| 8d | Thomas KG, Thomas MR, York EB, Dupras DM, Schultz HJ, Kolars JC: Teaching evidence-based medicine to internal medicine residents: The efficacy of conferences versus small-group discussion. Teach Learn Med 2005, 17:130-135. |
| 8e | Shuval K, Berkovits E, Netzer D, Hekselman I, Linn S, Brezis M, Reis S:Evaluating the impact of an evidence-based medicine educational intervention on primary care doctors' attitudes, knowledge and clinical behaviour: A controlled trial and before and after study. Journal of Evaluation in Clinical Practice 2007, 13(4):581-598. |
| 9a | Carlock D, Anderson J: Teaching and assessing the database searching skills of student nurses. Nurse Educ 2007, 32:251-255 |
| 9b | Webber M, Currin L, Groves N, Hay D, Fernando N: Social workers can e-learn: Evaluation of a pilot post-qualifying e-learning course in research methods and critical appraisal skills for social workers. Social Work Education 2010, 29:48-66. |
| 9c | Bradley DR, Rana GK, Martin PW, Schumacher RE: Real-time, evidence-based medicine instruction: A randomized controlled trial in a neonatal intensive care unit. J Med Libr Assoc 2002, 90:194-201. |
| 9d | Bennett KJ, Sackett DL, Haynes RB, Neufeld VR, Tugwell P, Roberts R: A controlled trial of teaching critical appraisal of the clinical literature to medical students. J Am Med Asscoc 1987, 257:2451-2454. |
| 10a | Feldstein DA, Maenner MJ, Srisurichan R, Roach MA, Vogelman BS: Evidence-based medicine training during residency: A randomized controlled trial of efficacy. BMC Med Educ 2010, 10:59. |
| 10b | Gardois P, Calabrese R, Colombi N, Deplano A, Lingua C, Longo F, Villanacci MC, Miniero R, Piga A: Effectiveness of bibliographic searches performed by paediatric residents and interns assisted by librarians. A randomised controlled trial. Health Information and Libraries Journal 2011, 28:273-284. |
| 10c | Ilic D, Tepper K, Misso M :Teaching evidence-based medicine literature searching skills to medical students during the clinical years: a randomized controlled trial. Journal of the Medical Library Association 2012, 100(3): 190-196. |
| 11a | Hugenholtz NI, Schaafsma FG, Nieuwenhuijsen K, van Dijk FJ: Effect of an EBM course in combination with case method learning sessions: An RCT on professional performance, job satisfaction, and self-efficacy of occupational physicians. International Archives of Occupational & Environmental Health 2008, 82:107-115 |
| 11b | Bradley P, Oterholt C, Herrin J, Nordheim L, Bjorndal A: Comparison of directed and self-directed learning in evidence-based medicine: A randomised controlled trial. Med Educ. 2005, 39:1027-1035. |
| 11c | Fu C, Hodges B, Regehr G, Goldbloom D, Garfinkel P: Is a journal club effective for teaching critical appraisal skills? A controlled trial with residents in psychiatry. Academic Psychiatry 1999, 23:205-209 |
| 11d | Bennett S, Hoffmann T, Arkins M: A multi-professional evidence-based practice course improved allied health students’ confidence and knowledge. J Eval Clin Pract 2011,17(4): 635-639 |
| 12a | Edwards R, White M, Gray J, Fischbacher C: Use of a journal club and letter-writing exercise to teach critical appraisal to medical undergraduates. Med Educ 2001, 35:691-694. |
| 12b | Smith CA, Ganschow PS, Reilly BM, Evans AT, McNutt RA, Osei A, Saquib M, Surhabi S, Yadav S: Teaching residents evidence-based medicine skills: A controlled trial of effectiveness and assessment of durability. J Gen Intern Med 2000, 15:710-715. |
| 13a | Arlt SP, Heuwieser W: Training students to appraise the quality of scientific literature. Journal of Veterinary Medical Education 2011, 38:135-140. |
| 13b | Webber M, Currin L, Groves N, Hay D, Fernando N: Social workers can e-learn: Evaluation of a pilot post-qualifying e-learning course in research methods and critical appraisal skills for social workers. Social Work Education 2010, 29:48-66. |
| 13c | Tomatis C, Taramona C, Rizo-Patron E, Hernandez F, Rodriguez P, Piscoya A, Gonzales E, Gotuzzo E, Heudebert G, Centor RM, Estrada CA: Evidence-based medicine training in a resource-poor country, the importance of leveraging personal and institutional relationships. Journal of Evaluation in Clinical Practice 2011, 17:644-650. |
| 14a | Badgett, RG, Paukert, JL, Levy, LS: Teaching clinical informatics to third-year medical students: Negative results from two controlled trials. BMC Med Educ 2001, 1: 3 |
| 14b | Carlock D, Anderson J: Teaching and assessing the database searching skills of student nurses. Nurse Educ 2007, 32:251-255. |
| 14c | Bradley P, Oterholt C, Herrin J, Nordheim L, Bjorndal A: Comparison of directed and self-directed learning in evidence-based medicine: A randomised controlled trial. Med Educ 2005, 39:1027-1035. |
| 15a | Bradley P, Oterholt C, Herrin J, Nordheim L, Bjorndal A: Comparison of directed and self-directed learning in evidence-based medicine: A randomised controlled trial. Med Educ 2005, 39:1027-1035. |
| 15b | McLeod RS, MacRae HM, McKenzie ME, Victor JC, Brasel KJ: Evidence Based Reviews in Surgery Steering Committee. A moderated journal club is more effective than an internet journal club in teaching critical appraisal skills: Results of a multicenter randomized controlled trial. J Am Coll Surg 2010, 211:769-776. |
| 15c | Srinivasan M, Weiner M, Breifeld PP, Brahmi F, Dickerson KL, Weiner G: Early introduction of an evidence-based medicine course to preclinical medical students. JGIM 2002, 17(1): 58-65. |
| 16a | Gardois P, Calabrese R, Colombi N, Deplano A, Lingua C, Longo F, Villanacci MC, Miniero R, Piga A: Effectiveness of bibliographic searches performed by paediatric residents and interns assisted by librarians. A randomised controlled trial. Health Information and Libraries Journal 2011, 28:273-284. |
| 16b | Abbott RD, O’Donnell J, Hawkins JD, Hill KG, Kosterman R, Catalano RF: Changing teaching practices to promote achievement and bonding to school. American Journal of Orthopsychiatry 1998, 68:542–552. |
| 17a | Saunders RP, Ward D, Felton GM, Dowda M, Pate RR: Examining the link between program implementation and behavior outcomes I the lifestyle education for activity program (LEAP). Evaluation and Program Planning 2006, 29: 352-364. |
| 17b | Resnicow K, Davis M, Smith M, Lararus-Yaroch A, Baranowski T, Baranowski J: How best to measure implementation of school health curricula: A comparison of three measures. Health Education Research 1998, 13(2):239–250. |
| 17c | Domitrovich CE, Greenberg M: The study of implementation: current findings from effective programs that prevent mental disorders in school-aged children. Journal of Education and Psychological Consultation 2000, 11(2): 193-221. |
